# Supplementary material for: Urolithin and Reduced Urolithin Derivatives as Potent Inhibitors of Tyrosinase and Melanogenesis: Importance of the 4-Substituted Resorcinol Moiety
Source: Int J Mol Sci. 2021 May 25;22(11):5616. doi: 10.3390/ijms22115616 (PMC8199067; doi:10.3390/ijms22115616)
Supplement: Supplementary file 1 [file ijms-22-05616-s001.zip › ijms-1218589-supplementary.pdf]

Supplementary Information

Urolithin and reduced urolithin derivatives as potent inhibitors of tyrosinase and melanogenesis: importance of the 4-substituted resorcinol moiety

Sanggwon Lee[a,§], Heejeong Choi[a,§], Yujin Park[a], Hee Jin Jung[a], Sultan Ullah[b], Inkyu Choi[a], Dongwan Kang[a], Chaeun Park[a], Il Young Ryu[a], Yeongmu Jeong[a], YeJi Hwang[a], Sojeong Hong[a], Pusoon Chun[c], Hyung Ryong Moon[a,*]

[a]*College of Pharmacy, Pusan National University, Busan 46241, South Korea*

[b]*Department of Molecular Medicine, The Scripps Research Institute, Florida 33458, USA*

[c]*College of Pharmacy and Inje Institute of Pharmaceutical Sciences and Research, Inje University, Gimhae, Gyeongnam 50834, South Korea*

Contents

^{1}H NMR spectrum of compound **1a** 4

^{13}C NMR spectrum of compound **1a** 5

Mass spectrum of compound **1a** 6

^{1}H NMR spectrum of compound **1b** 7

^{13}C NMR spectrum of compound **1b** 8

High resolution Mass spectrum of compound **1b** 9

^{1}H NMR spectrum of compound **1c** 10

^{13}C NMR spectrum of compound **1c** 11

Mass spectrum of compound **1c** 12

^{1}H NMR spectrum of compound **1d** 13

^{13}C NMR spectrum of compound **1d** 14

Mass spectrum of compound **1d** 15

^{1}H NMR spectrum of compound **1e** 16

^{13}C NMR spectrum of compound **1e** 17

Mass spectrum of compound **1e** 18

^{1}H NMR spectrum of compound **1f** 19

^{13}C NMR spectrum of compound **1f** 20

Mass spectrum of compound **1f** 21

^{1}H NMR spectrum of compound **1g** 22

^{13}C NMR spectrum of compound **1g** 23

Mass spectrum of compound **1g** 24

^{1}H NMR spectrum of compound **1h** 25

^{13}C NMR spectrum of compound **1h** 26

Mass spectrum of compound **1h** 27

^{1}H NMR spectrum of compound **1i** 28

^{13}C NMR spectrum of compound **1i** 29

High resolution Mass spectrum of compound **1i** 30

^{1}H NMR spectrum of compound **1j** 31

^{13}C NMR spectrum of compound **1j** 32

High resolution Mass spectrum of compound **1j** 33

^{1}H NMR spectrum of compound **2a** 34

^{13}C NMR spectrum of compound **2a** 35
Mass spectrum of compound **2a** 36
High resolution Mass spectrum of compound **2a** 37
^{1}H NMR spectrum of compound **2b** 38
^{13}C NMR spectrum of compound **2b** 39
Mass spectrum of compound **2b** 40
^{1}H NMR spectrum of compound **2c** 41
^{13}C NMR spectrum of compound **2c** 42
Mass spectrum of compound **2c** 43
^{1}H NMR spectrum of compound **2d** 44
^{13}C NMR spectrum of compound **2d** 45
Mass spectrum of compound **2d** 46
^{1}H NMR spectrum of compound **2e** 47
^{13}C NMR spectrum of compound **2e** 48
Mass spectrum of compound **2e** 49
^{1}H NMR spectrum of compound **2f** 50
^{13}C NMR spectrum of compound **2f** 51
Mass spectrum of compound **2f** 52
^{1}H NMR spectrum of compound **2g** 53
^{13}C NMR spectrum of compound **2g** 54
Mass spectrum of compound **2g** 55
^{1}H NMR spectrum of compound **2h** 56
^{13}C NMR spectrum of compound **2h** 57
Mass spectrum of compound **2h** 58
^{1}H NMR spectrum of compound **2i** 59
^{13}C NMR spectrum of compound **2i** 60
Mass spectrum of compound **2i** 61
Raw data for IC_{50} values of **1c**, **1h**, **2a** and kojic acid........ 62
Diagram of IC_{50} values for **1c**, **1h**, **2a** and kojic acid........ 63

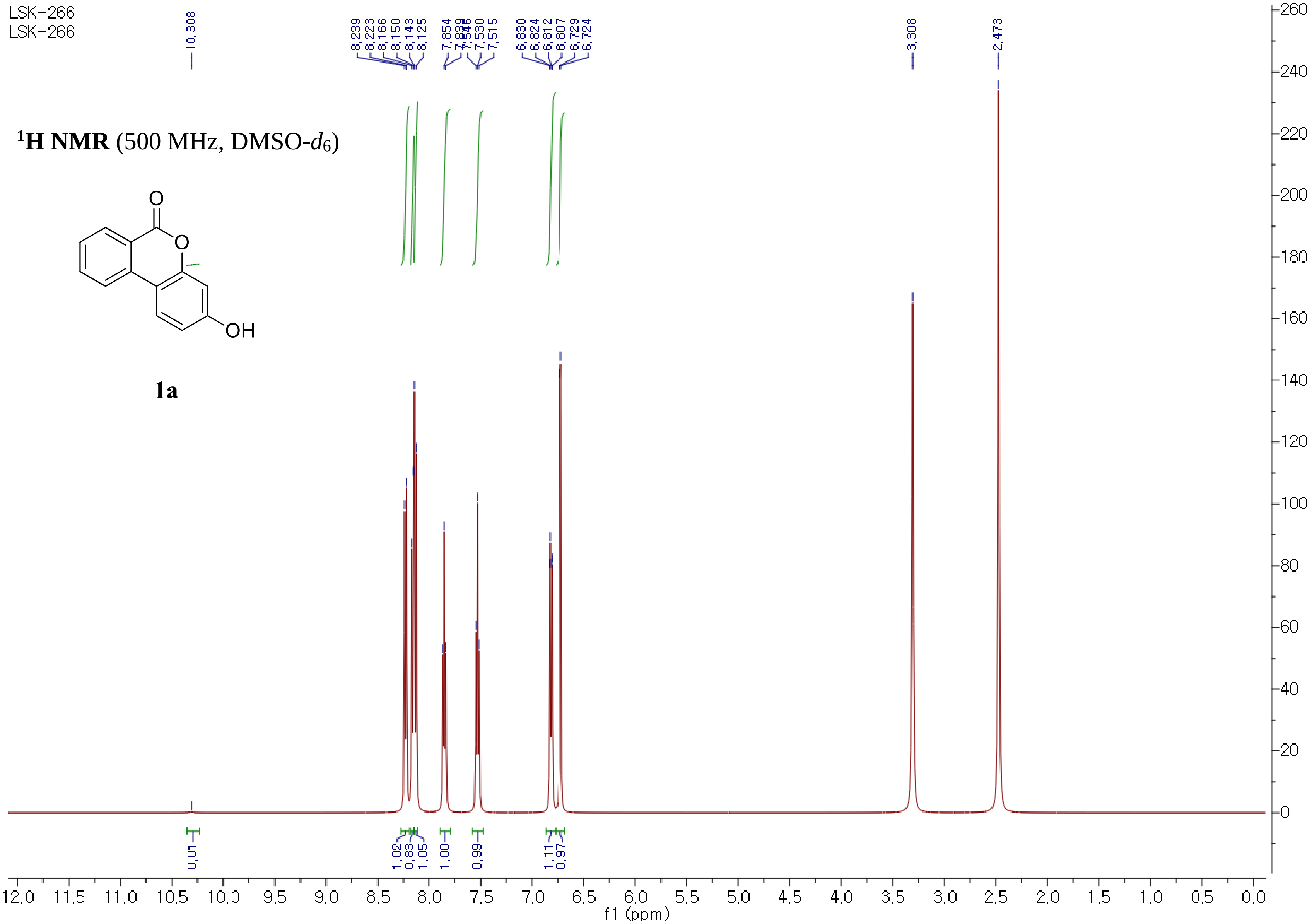
LSK-266
LSK-266
10.308
8.239
8.223
8.166
8.150
8.143
8.125
7.854
7.839
7.546
7.530
7.515
6.830
6.824
6.812
6.807
6.729
6.724
3.308
2.473
^{1}H NMR (500 MHz, DMSO-d_6)
O
O
OH
1a
0.01
1.02
0.83
1.05
1.00
0.99
1.11
0.97
f1 (ppm)

^{13}C NMR (100 MHz, DMSO-d_6)

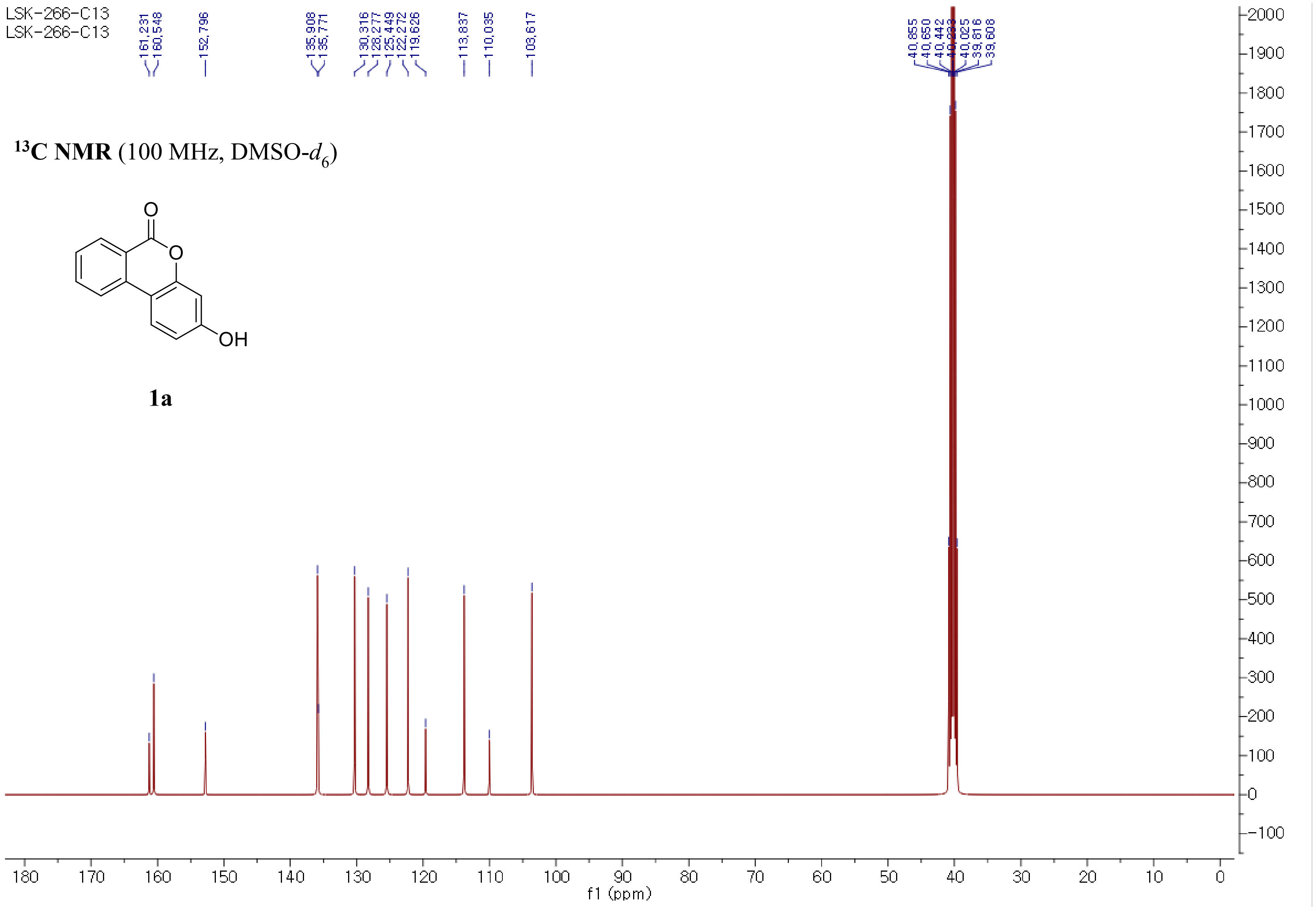

1a

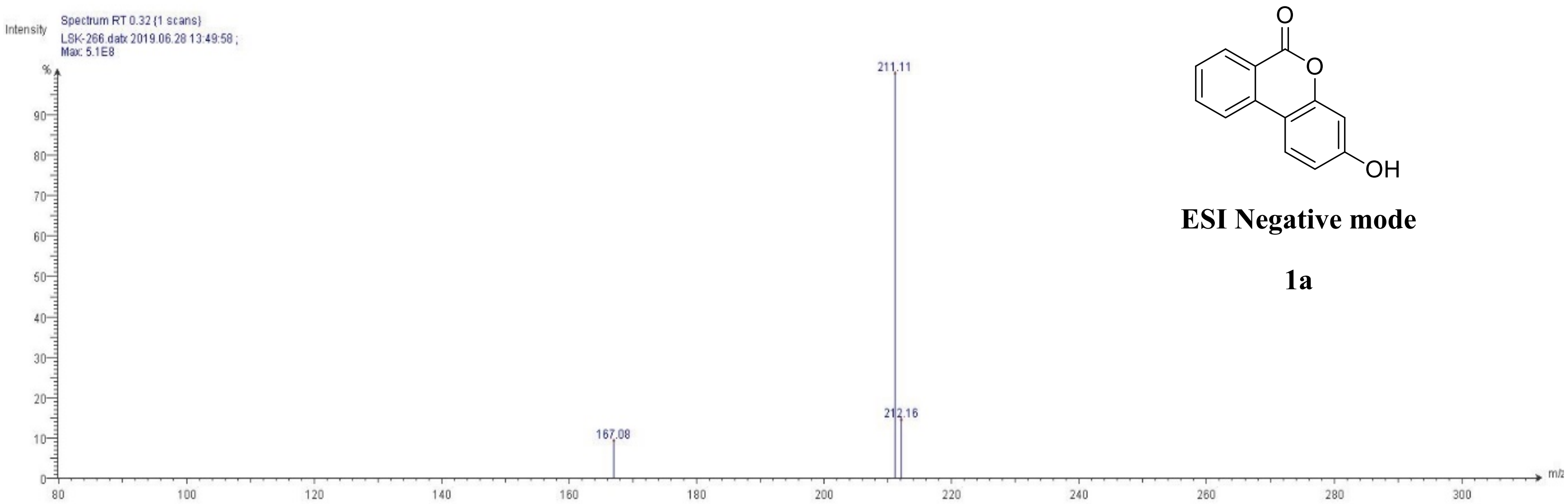

ESI Negative mode

1a

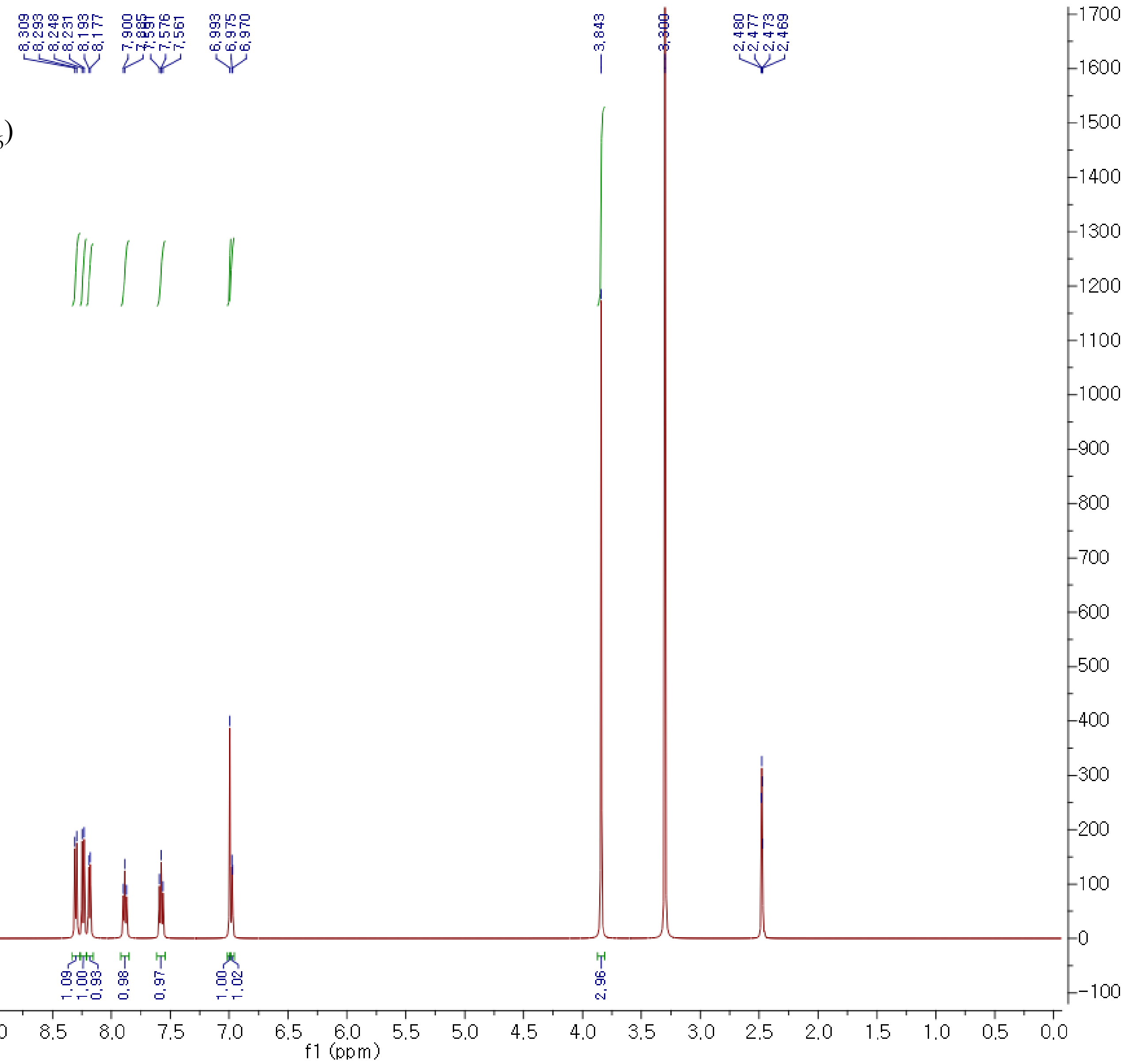

^{1}**H NMR** (500 MHz, DMSO-d_6)

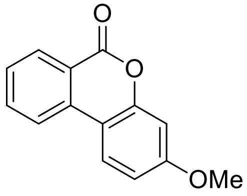

1b

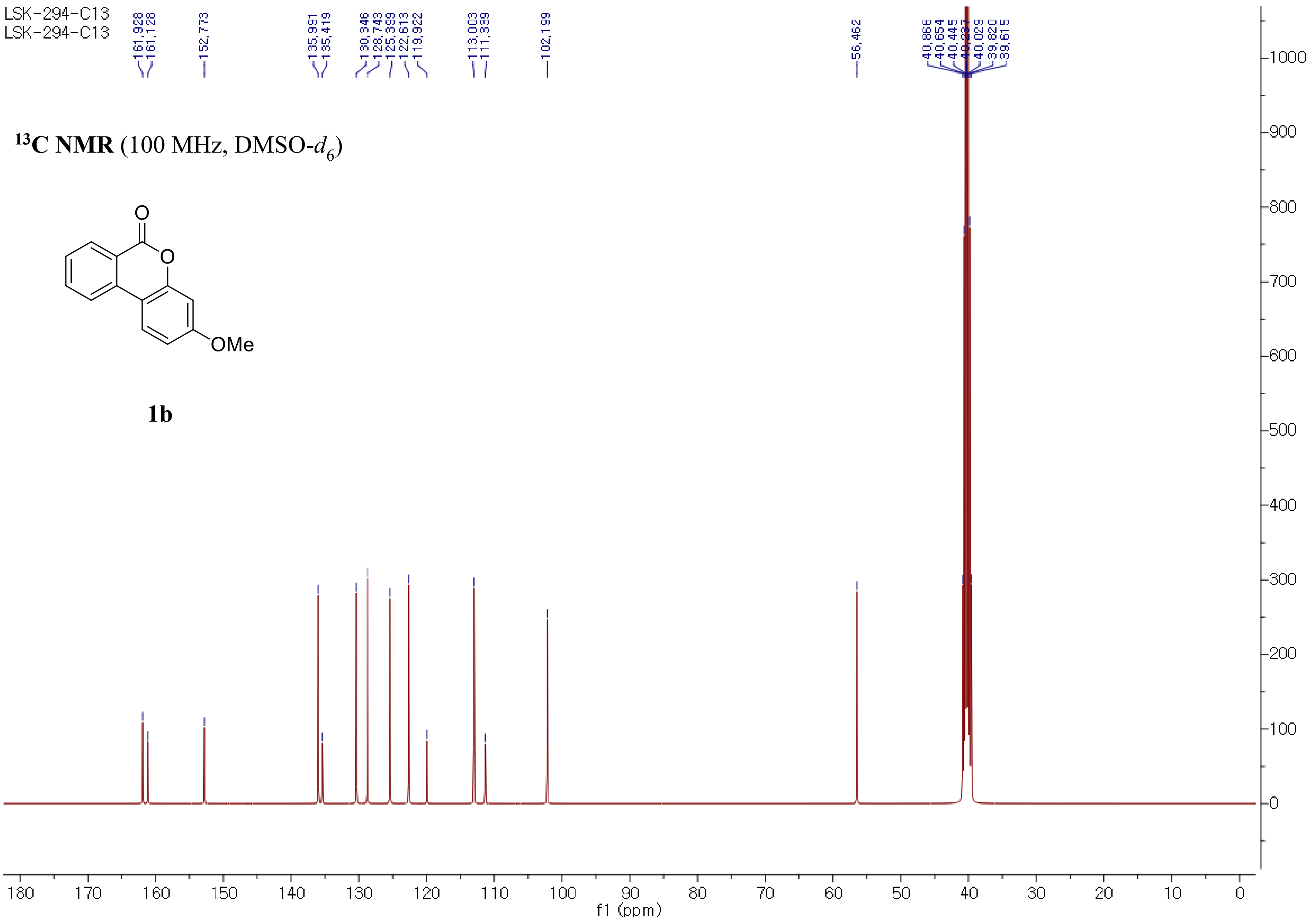
LSK-294-C13
LSK-294-C13
161.928
161.128
152.773
135.991
135.419
130.346
128.743
125.399
122.613
119.922
113.003
111.339
102.199
56.462
40.866
40.654
40.445
40.029
39.820
39.615
^{13}C NMR (100 MHz, DMSO-d_6)
O
O
OMe
1b
f1 (ppm)

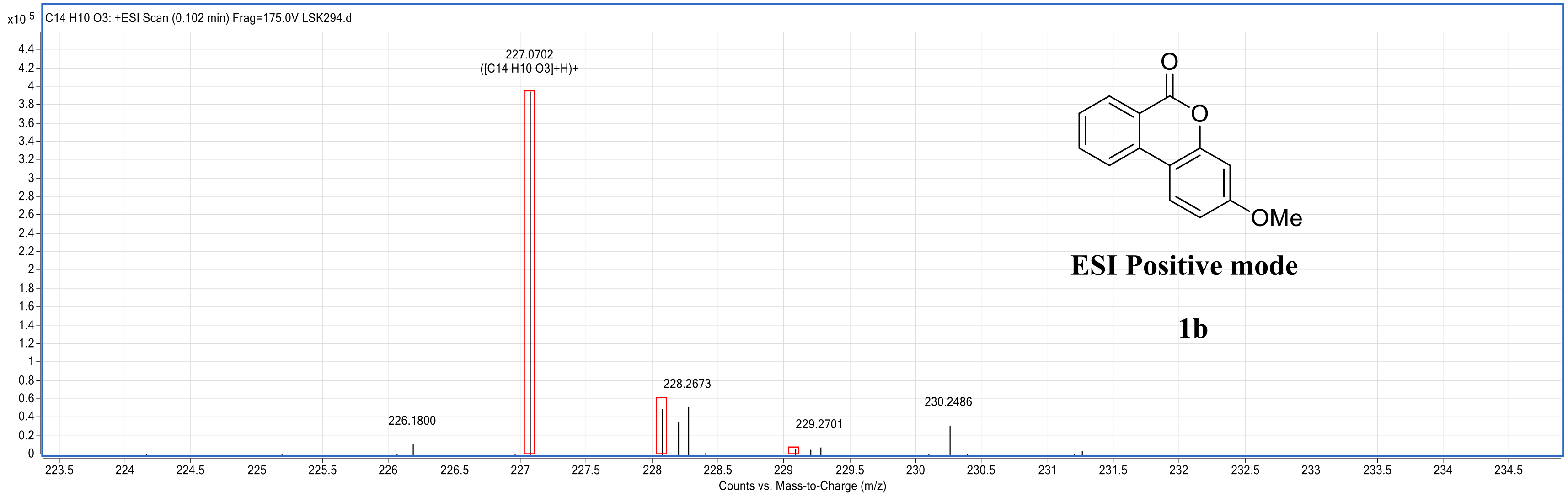
x10 5
C14 H10 O3: +ESI Scan (0.102 min) Frag=175.0V LSK294.d
227.0702
([C14 H10 O3]+H)+
226.1800
228.2673
229.2701
230.2486
OMe
O
ESI Positive mode
1b
Counts vs. Mass-to-Charge (m/z)

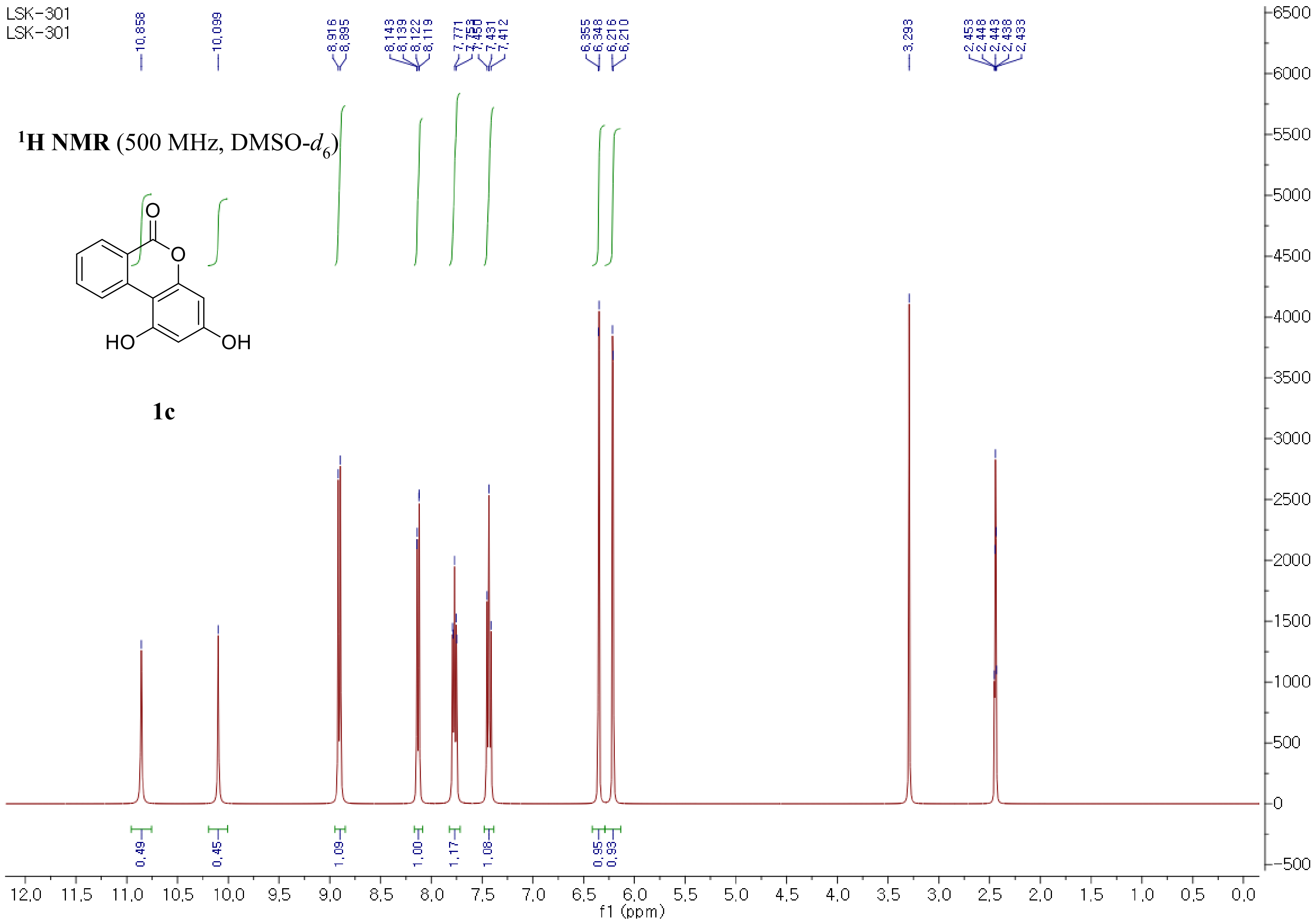
LSK-301
LSK-301
¹H NMR (500 MHz, DMSO-d6)
1c
10.858
10.099
8.916
8.895
8.143
8.139
8.122
8.119
7.771
7.431
7.412
6.355
6.348
6.216
6.210
3.293
2.453
2.448
2.443
2.438
2.433
0.49
0.45
1.09
1.00
1.17
1.08
0.95
0.93
f1 (ppm)

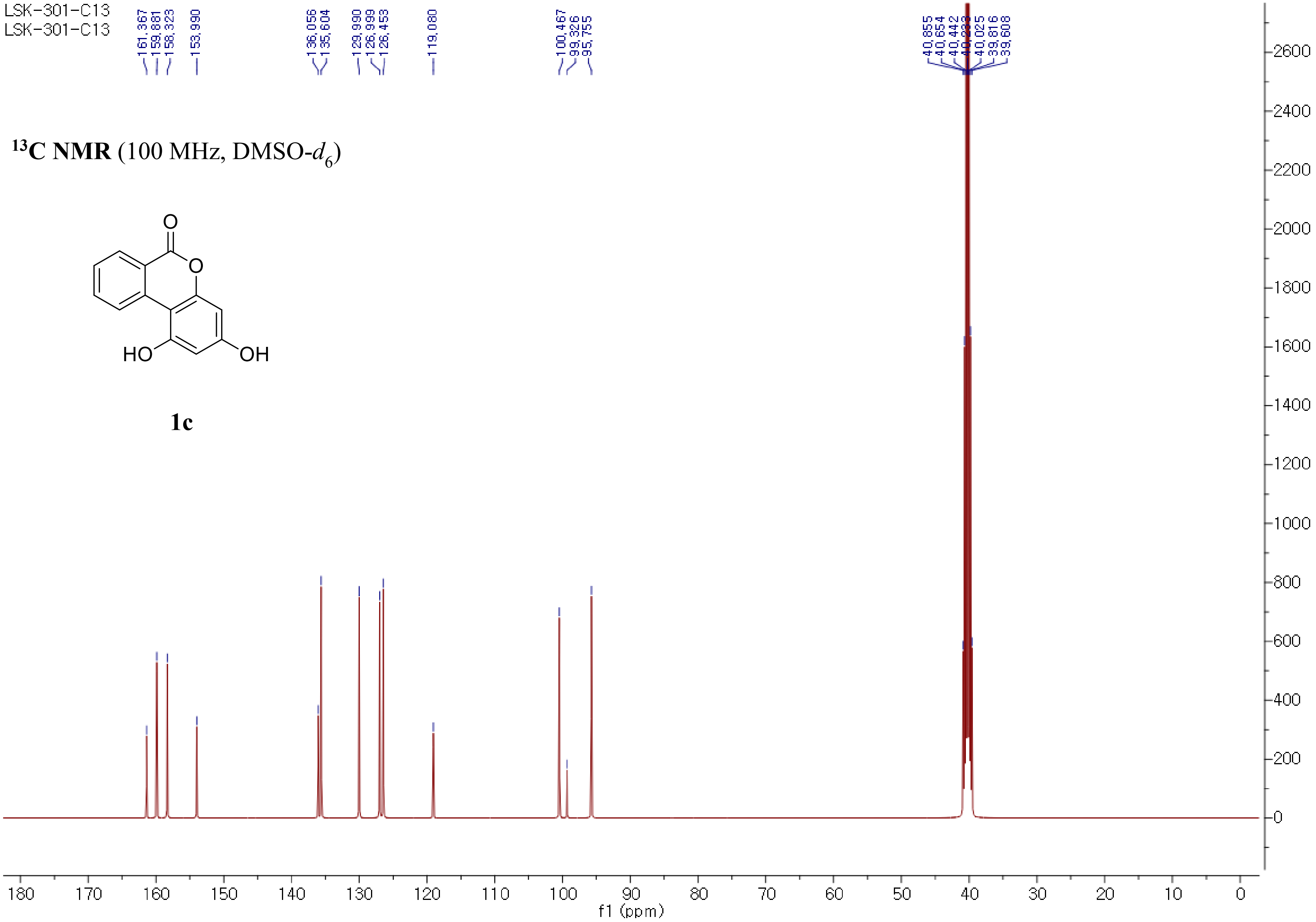

^{13}C NMR (100 MHz, DMSO-d_6)

1c

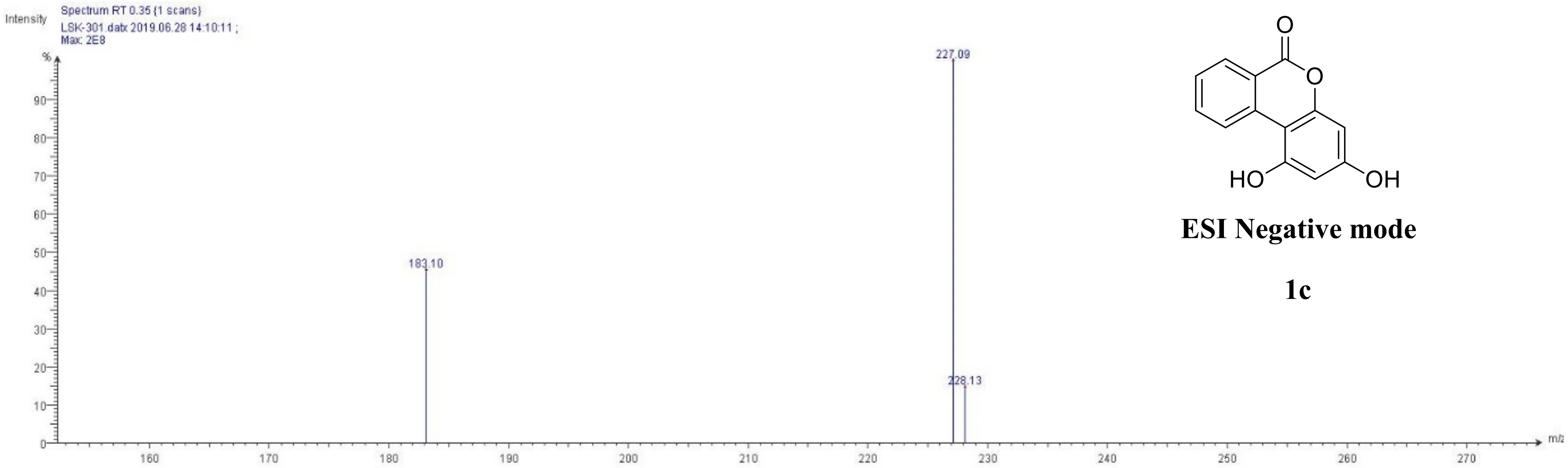

ESI Negative mode

1c

^{1}H NMR (500 MHz, DMSO-d_6)

1d

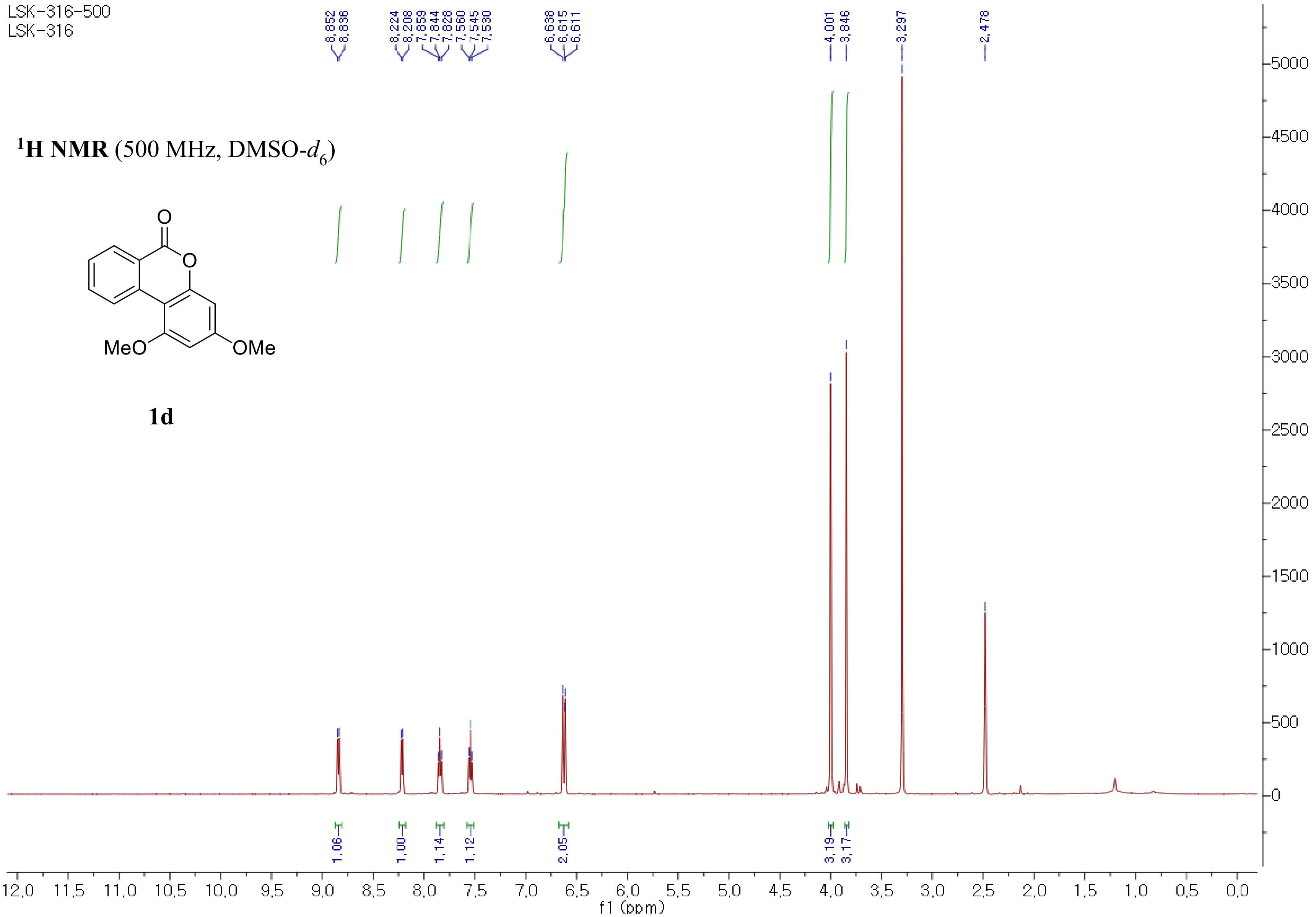

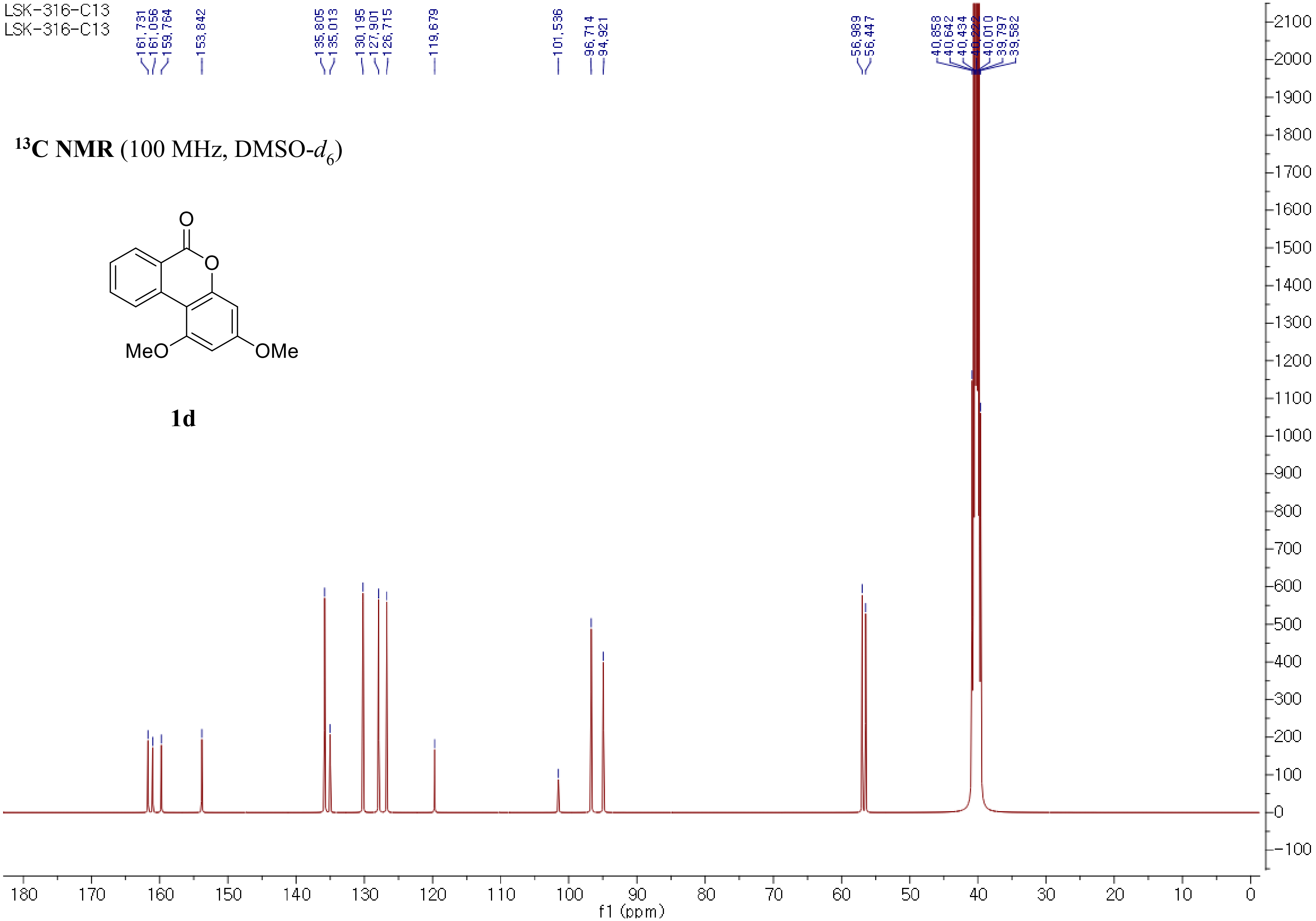

^{13}C NMR (100 MHz, DMSO-d_6)

1d

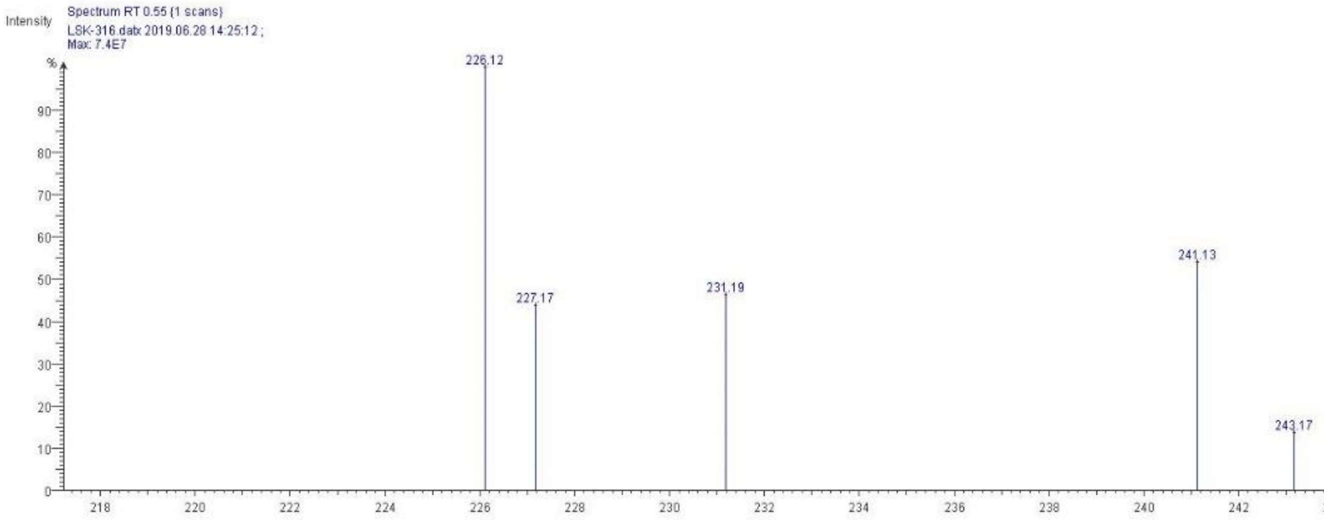

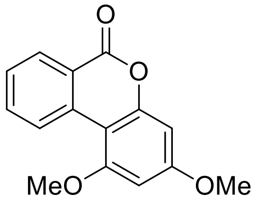

ESI Negative mode

1d

^{1}H NMR (500 MHz, DMSO-d_6)

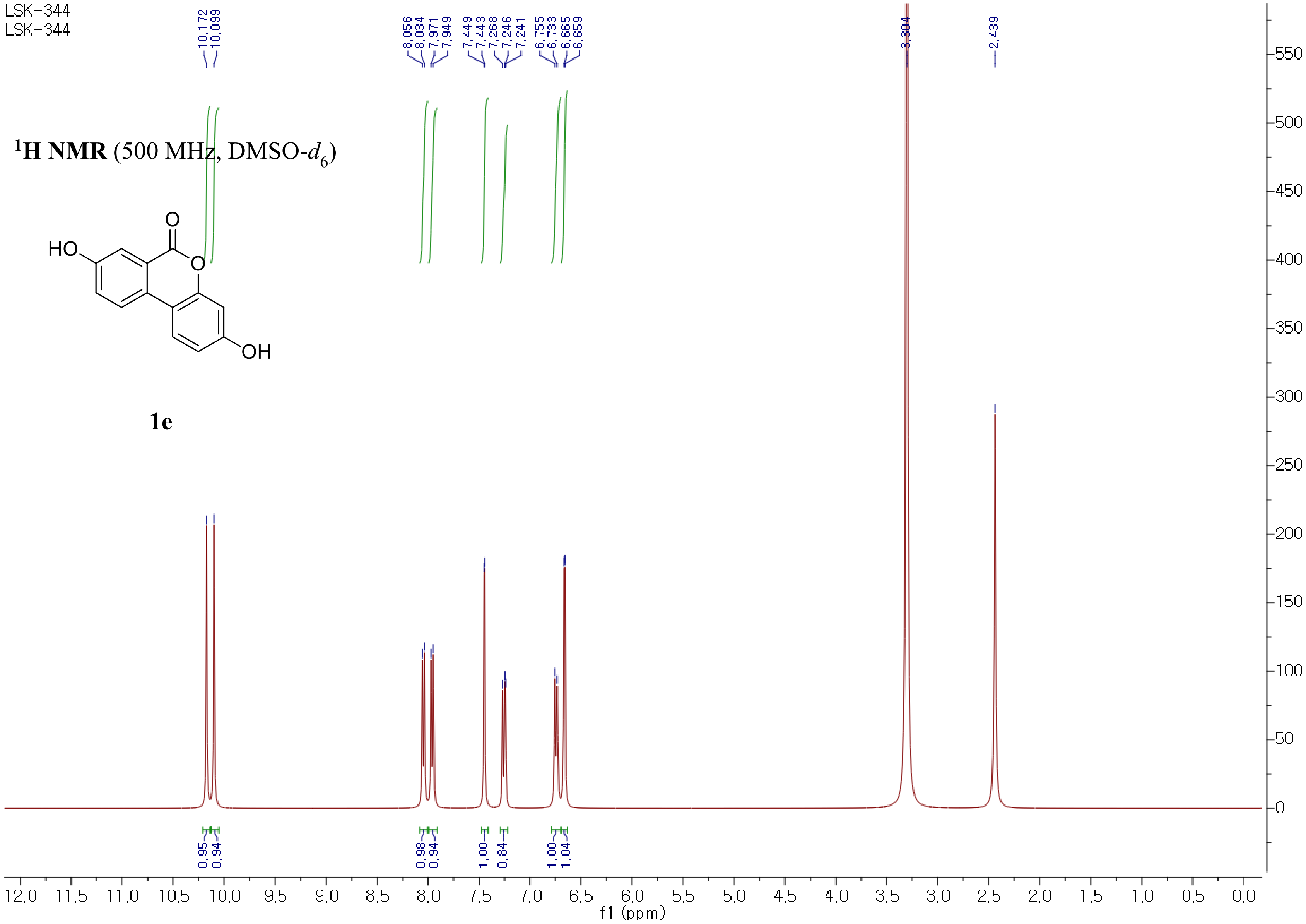

1e

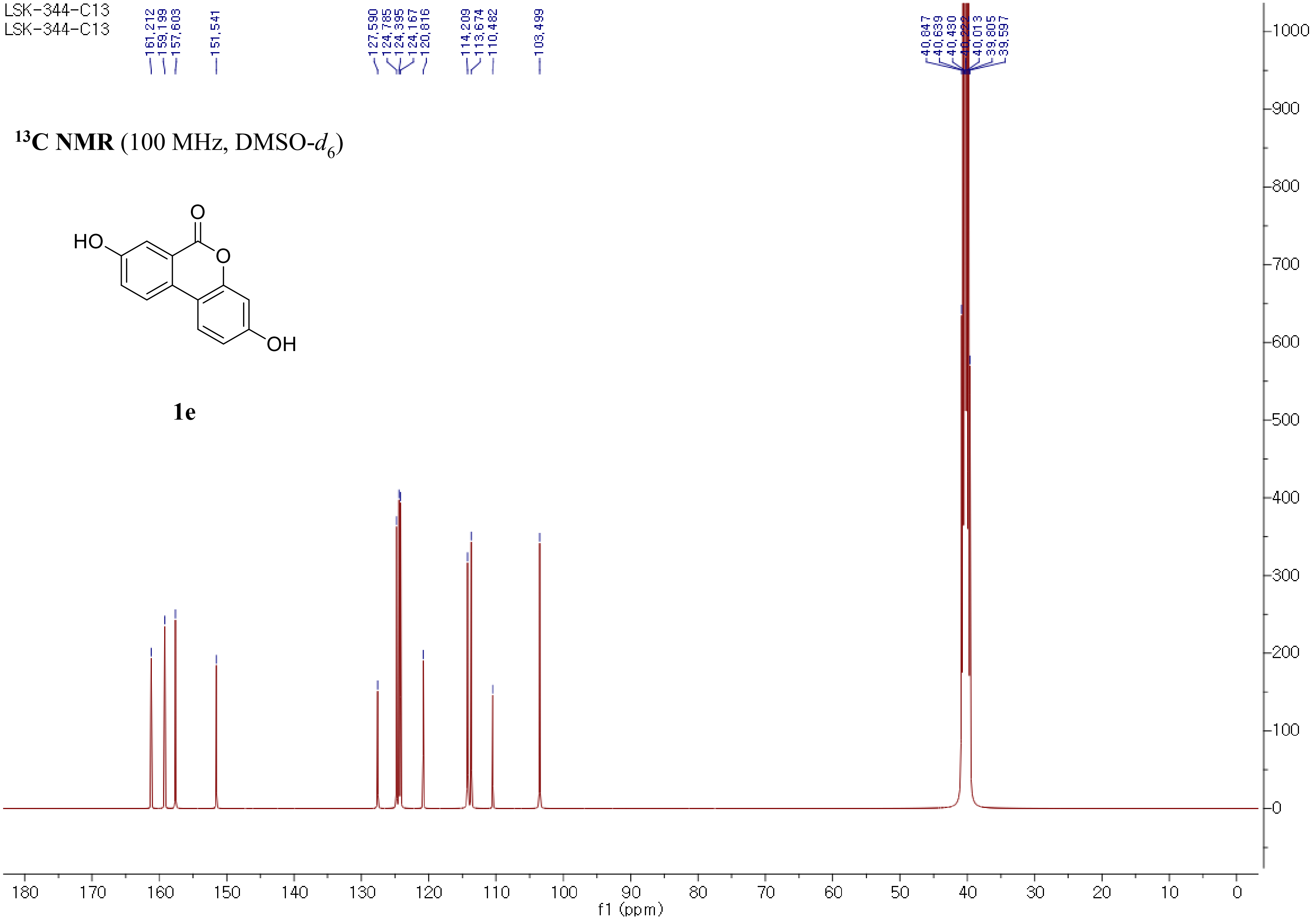
LSK-344-C13
LSK-344-C13
161.212
159.199
157.603
151.541
127.590
124.785
124.395
124.167
120.816
114.209
113.674
110.482
103.499
40.847
40.639
40.430
40.222
40.013
39.805
39.597
^{13}C NMR (100 MHz, DMSO-d_6)
HO
O
O
OH
1e
f1 (ppm)

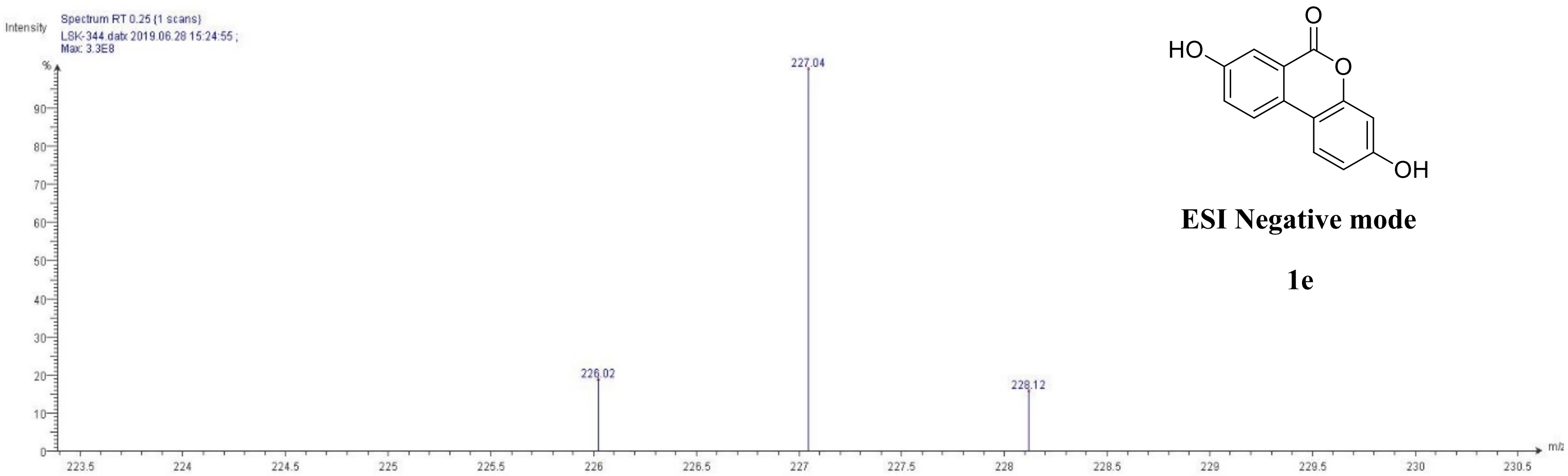

ESI Negative mode

1e

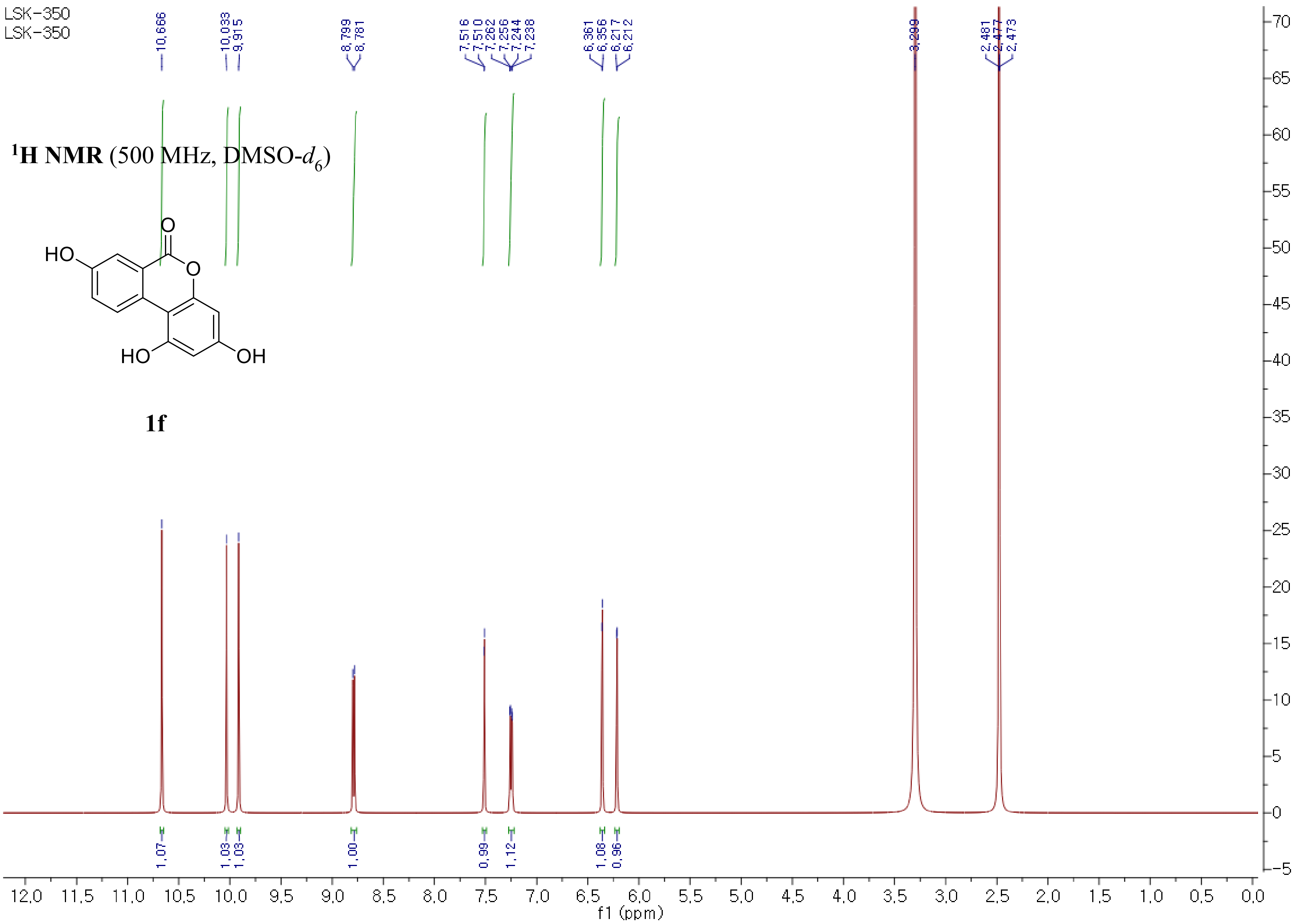
LSK-350
LSK-350
10.666
10.033
9.915
8.799
8.781
7.516
7.510
7.262
7.256
7.244
7.238
6.361
6.356
6.217
6.212
3.299
2.481
2.477
2.473
^{1}H NMR (500 MHz, DMSO-d_6)
O
HO
O
HO
OH
1f
1.07
1.03
1.03
1.00
0.99
1.12
1.08
0.96
f1 (ppm)

^{13}**C NMR** (100 MHz, DMSO-d_6)

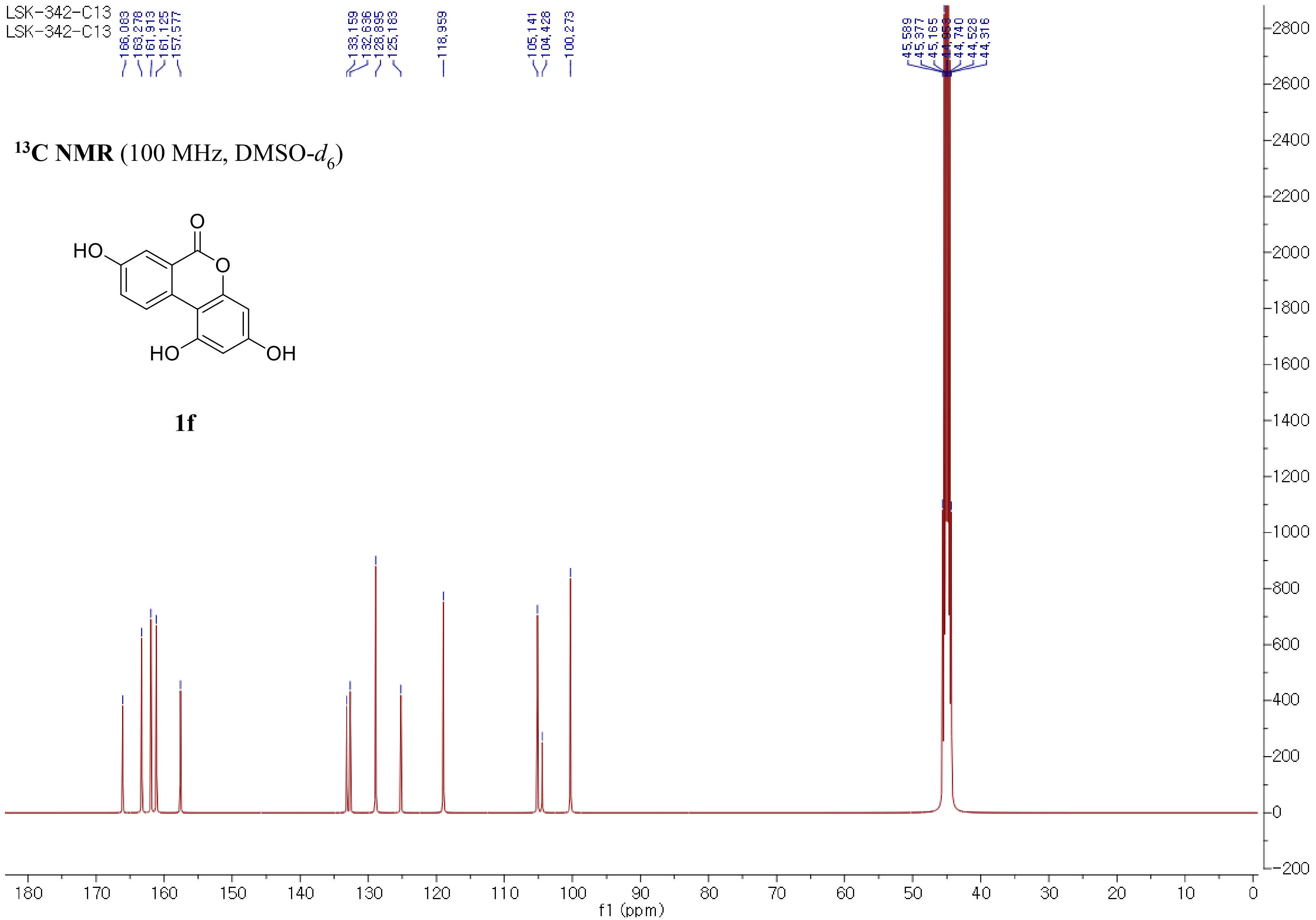

1f

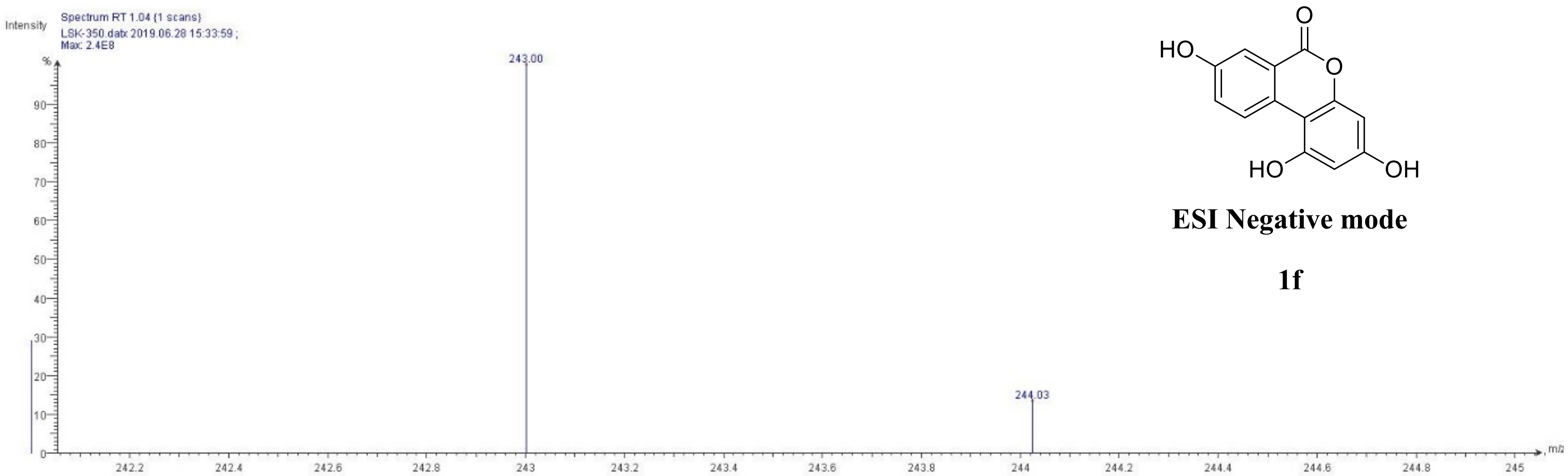

ESI Negative mode

1f

^{1}**H NMR** (500 MHz, DMSO-d_6)

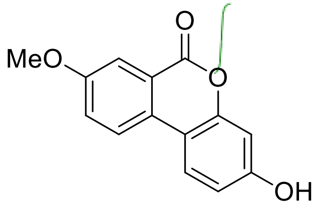

1g

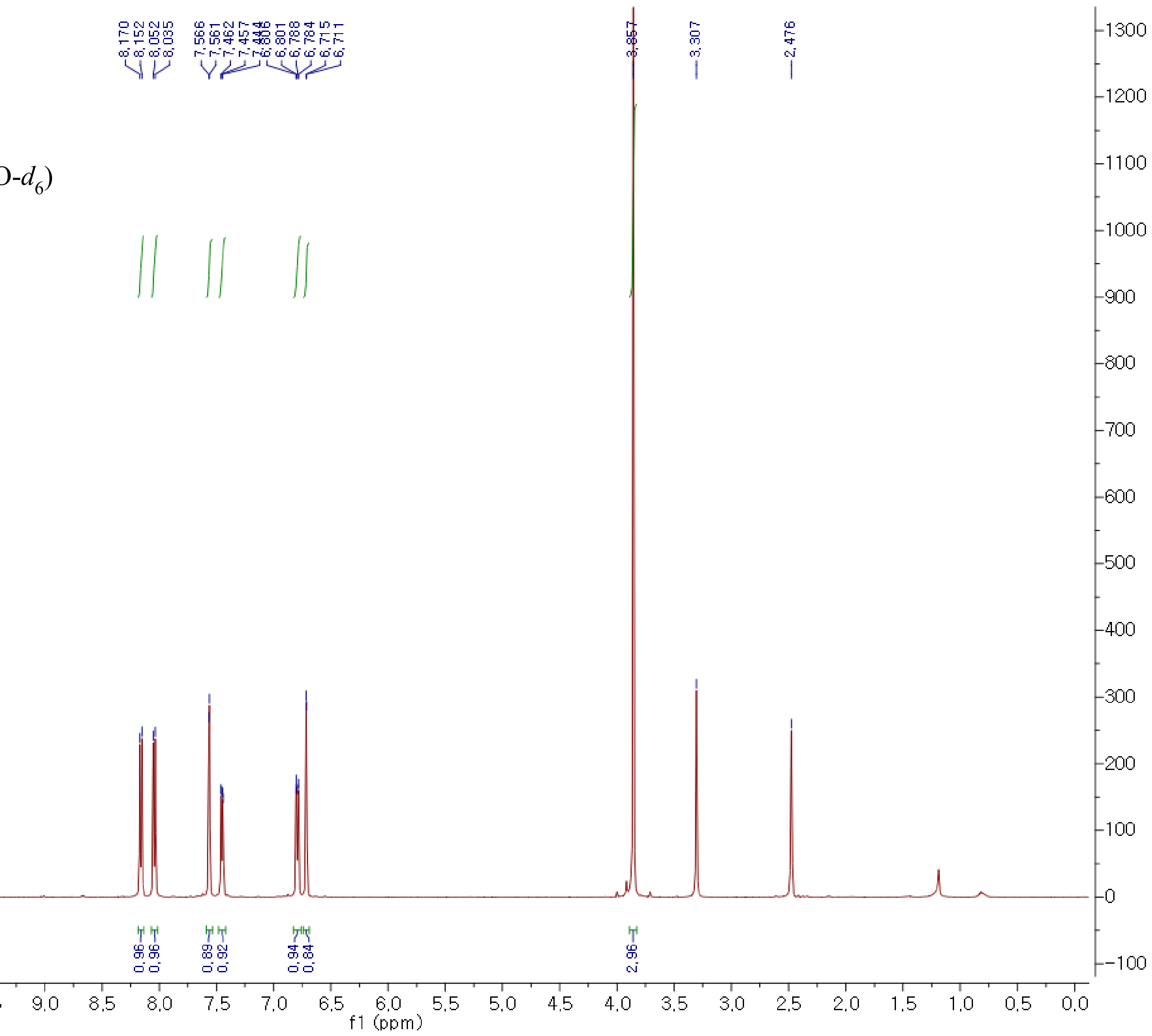

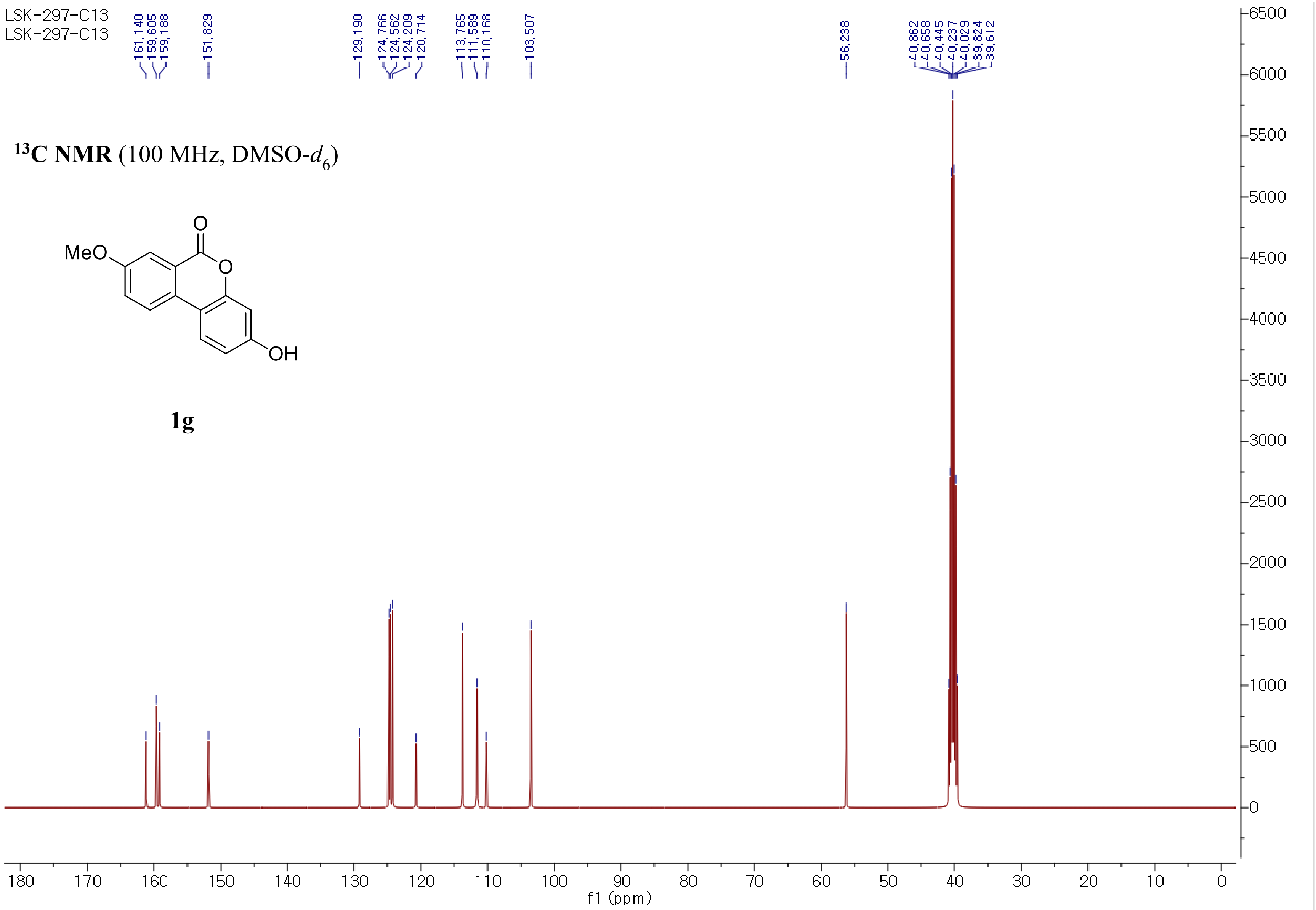
LSK-297-C13
LSK-297-C13
161.140
159.605
159.188
151.829
129.190
124.766
124.562
124.209
120.714
113.765
111.589
110.168
103.507
56.238
40.862
40.658
40.445
40.237
40.029
39.824
39.612
13C NMR (100 MHz, DMSO-d6)
MeO
O
O
OH
1g
f1 (ppm)

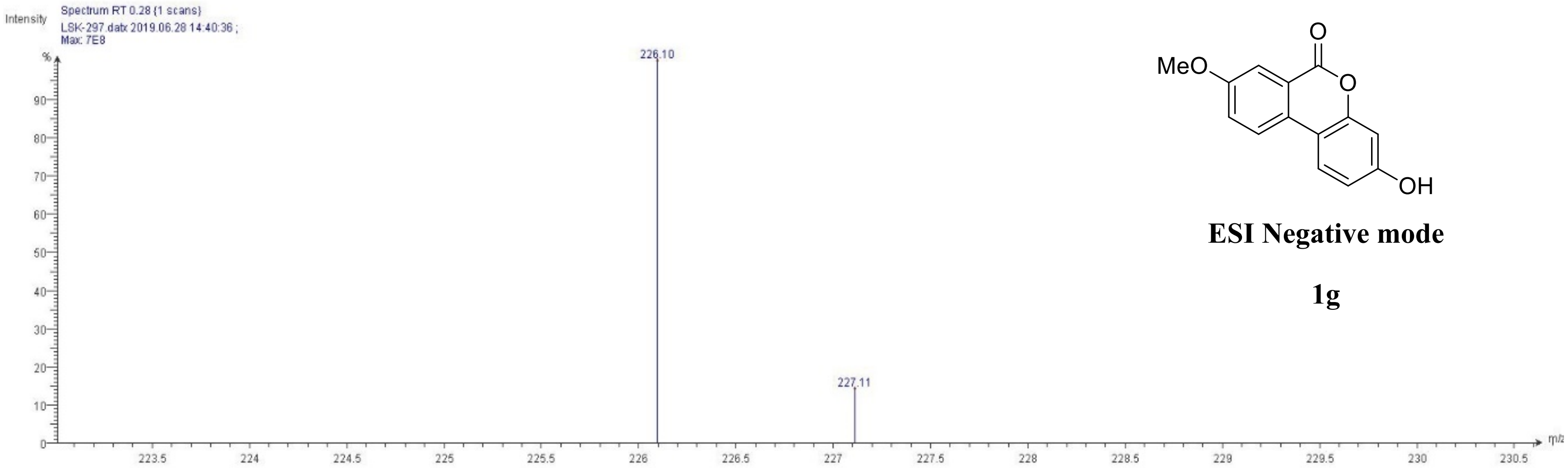

ESI Negative mode

1g

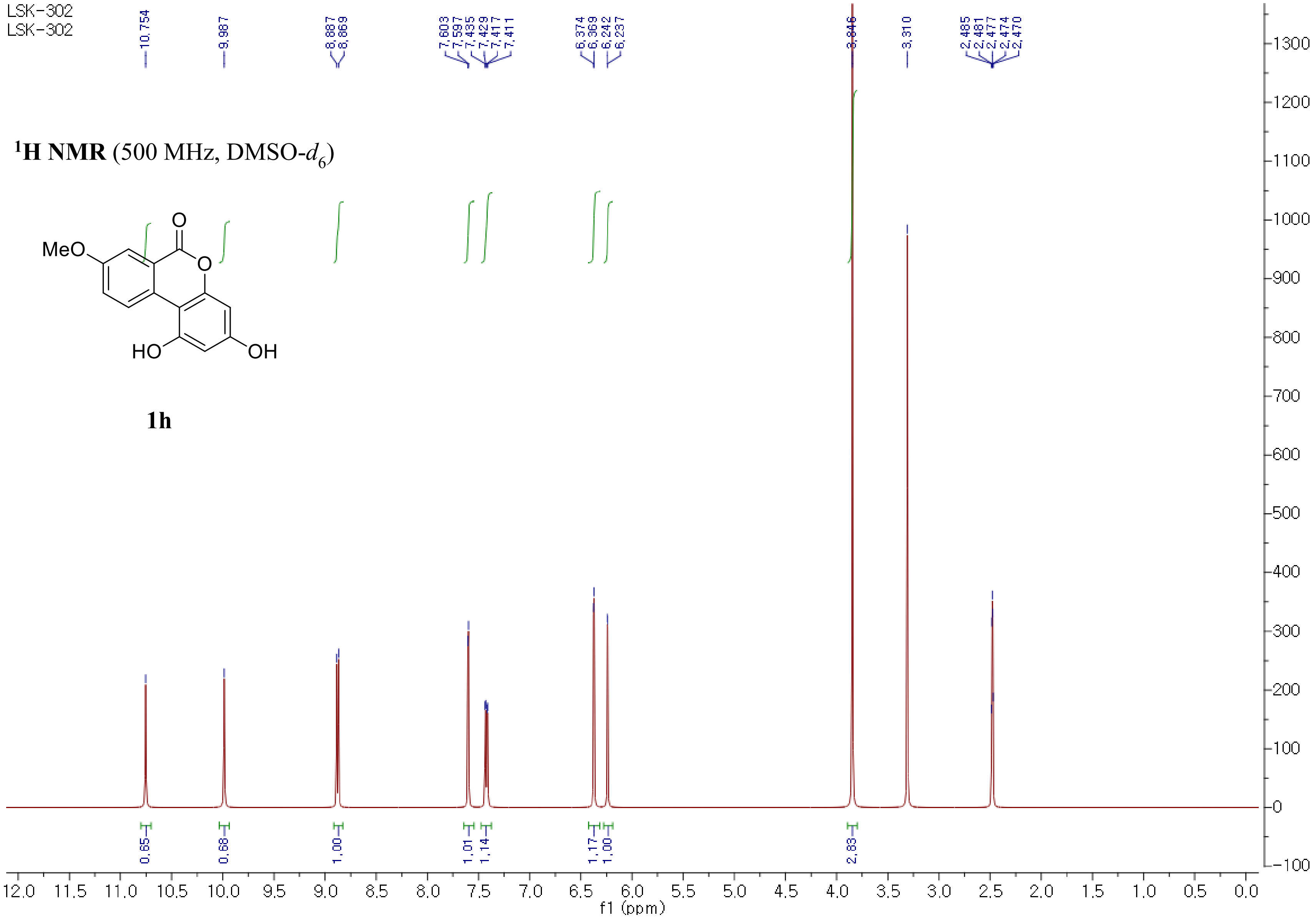
LSK-302
LSK-302
^{1}H NMR (500 MHz, DMSO-d_6)
MeO
O
O
HO
OH
1h
10.754
9.987
8.887
8.869
7.603
7.597
7.435
7.429
7.417
7.411
6.374
6.369
6.242
6.237
3.846
3.310
2.485
2.481
2.477
2.474
2.470
0.65
0.68
1.00
1.01
1.14
1.17
1.00
2.83
f1 (ppm)

^{13}C NMR (100 MHz, DMSO-d_6)

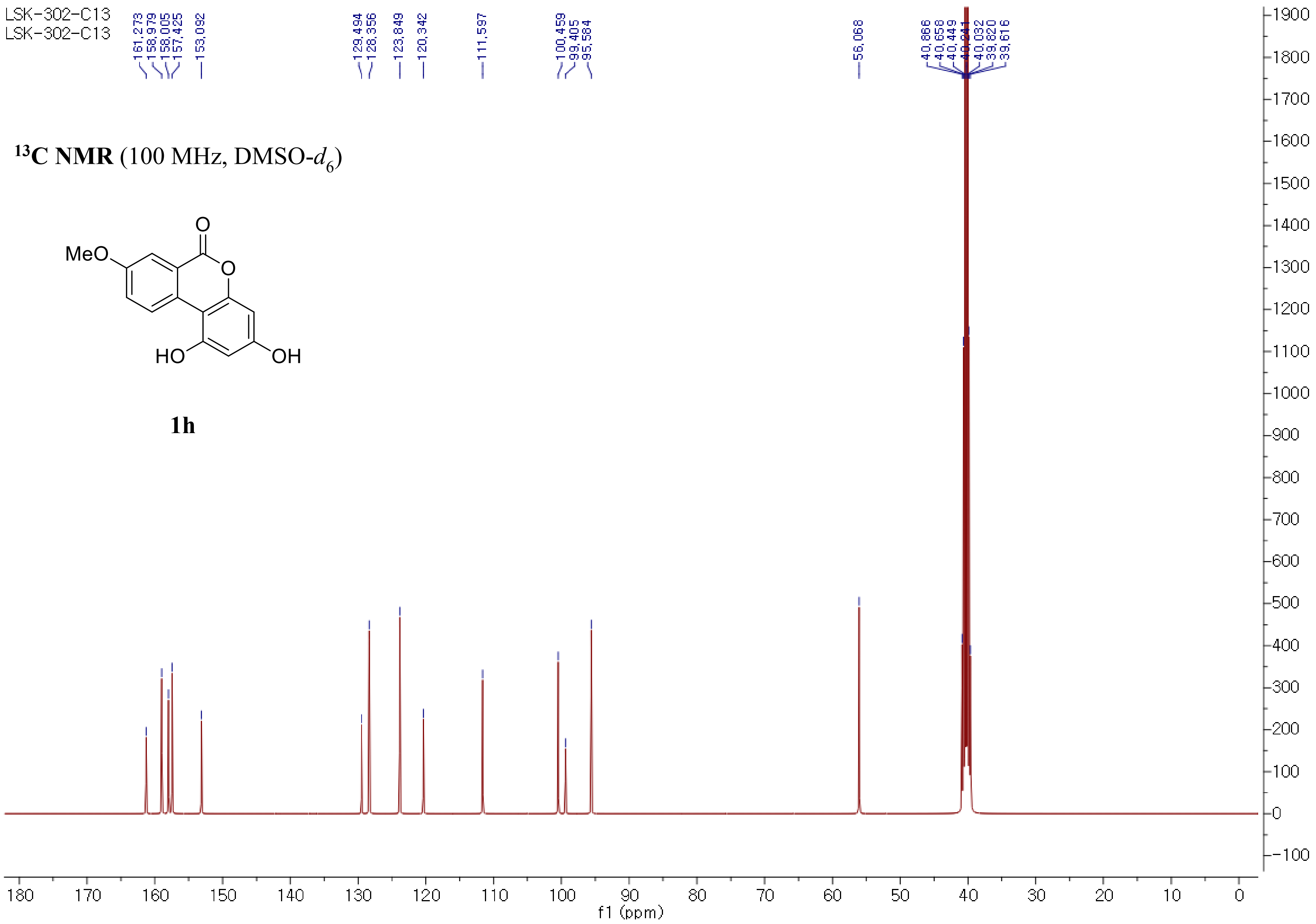

1h

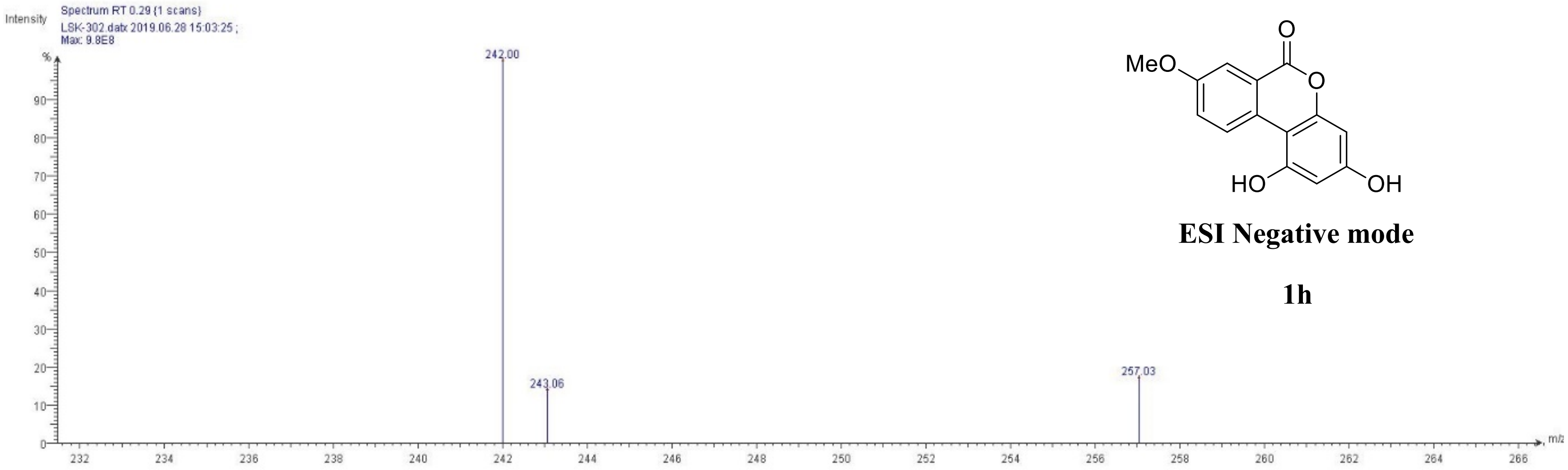

ESI Negative mode

1h

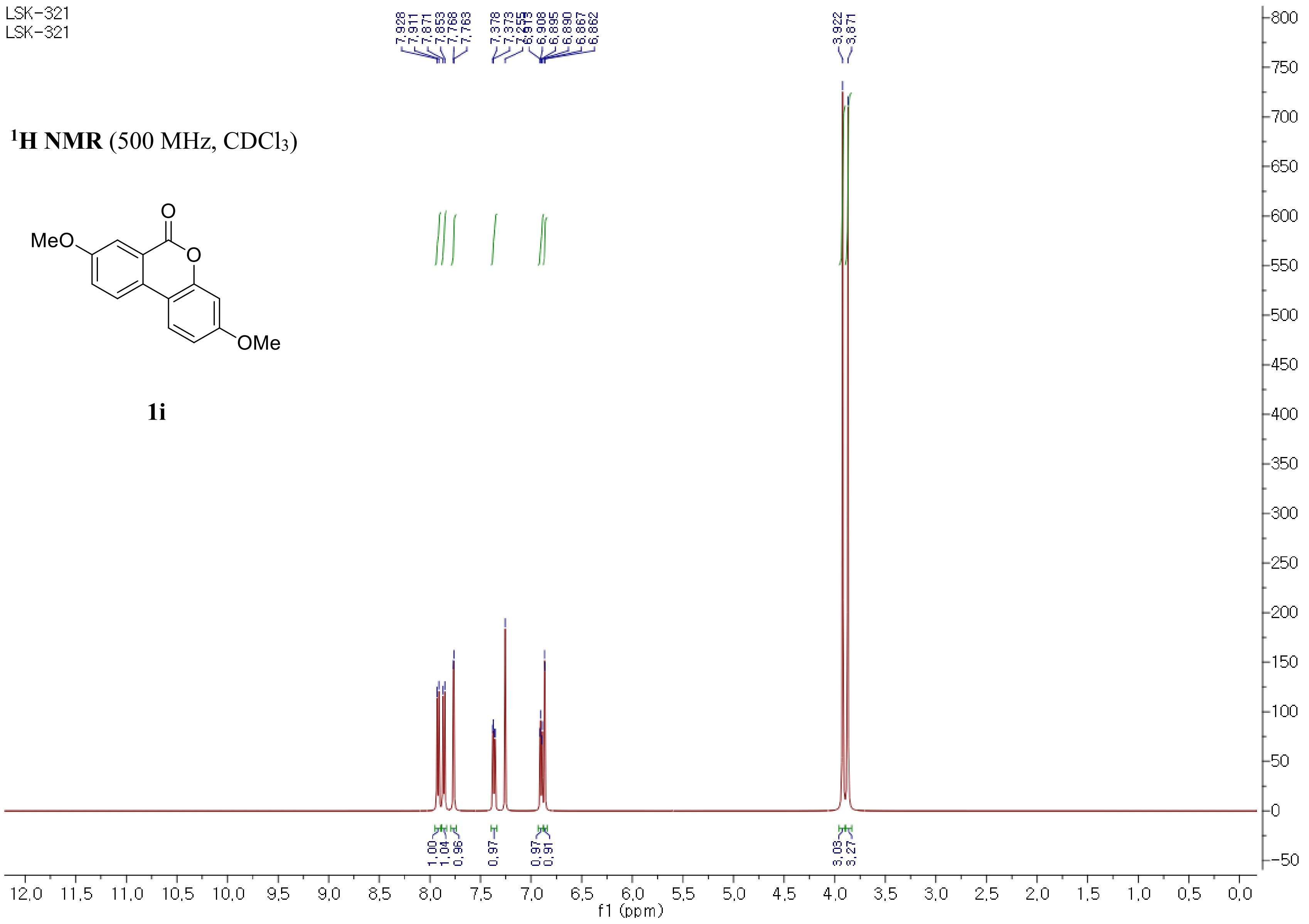
LSK-321
LSK-321
^{1}H NMR (500 MHz, CDCl$_3$)
MeO
O
O
OMe
1i
7.928 7.911 7.871 7.853 7.768 7.763
7.378 7.373 7.259 6.913 6.908 6.895 6.890 6.867 6.862
3.922 3.871
1.00 1.04 0.96 0.97 0.97 0.91 3.03 3.27
f1 (ppm)

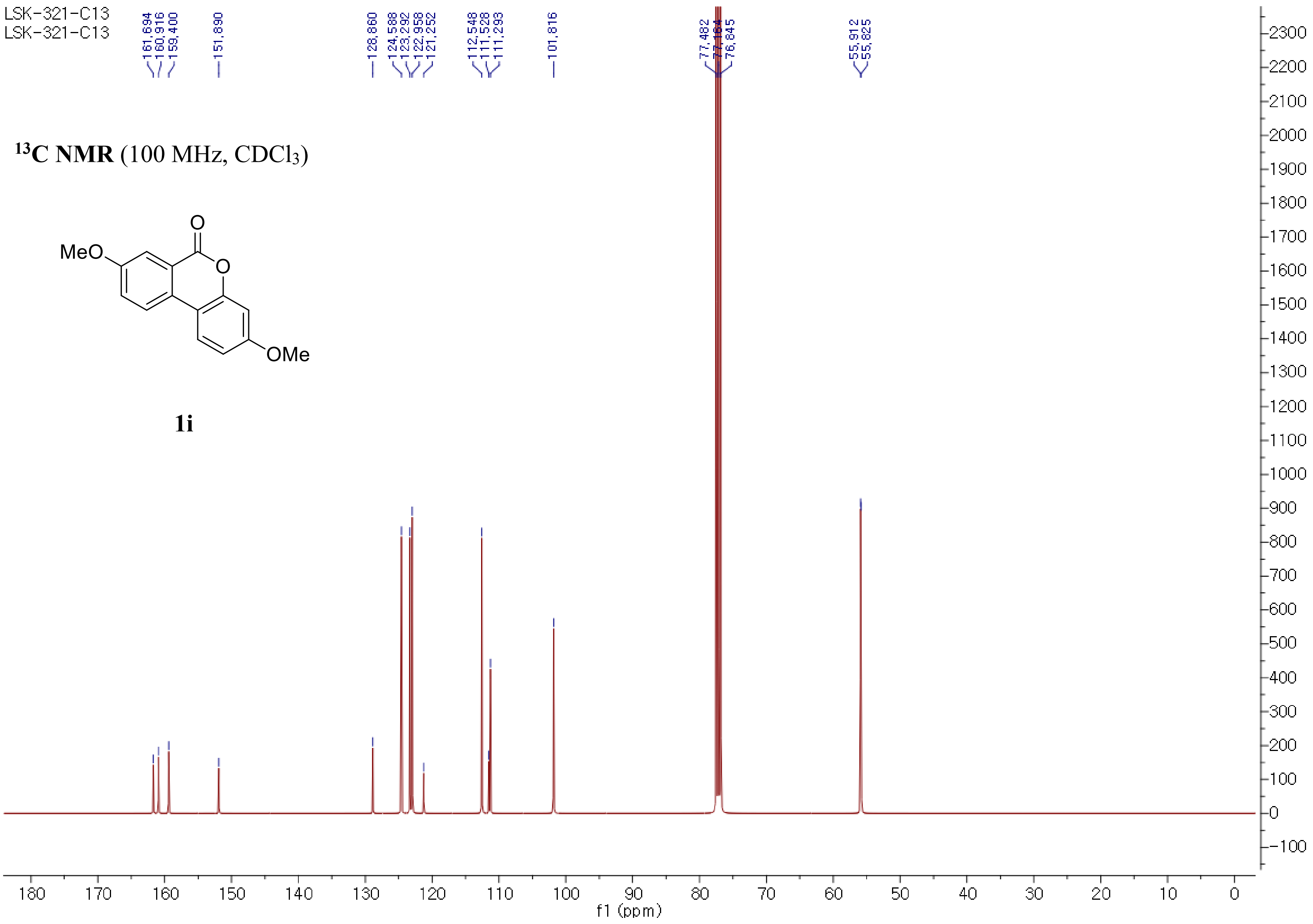

^{13}C NMR (100 MHz, CDCl$_3$)

1i

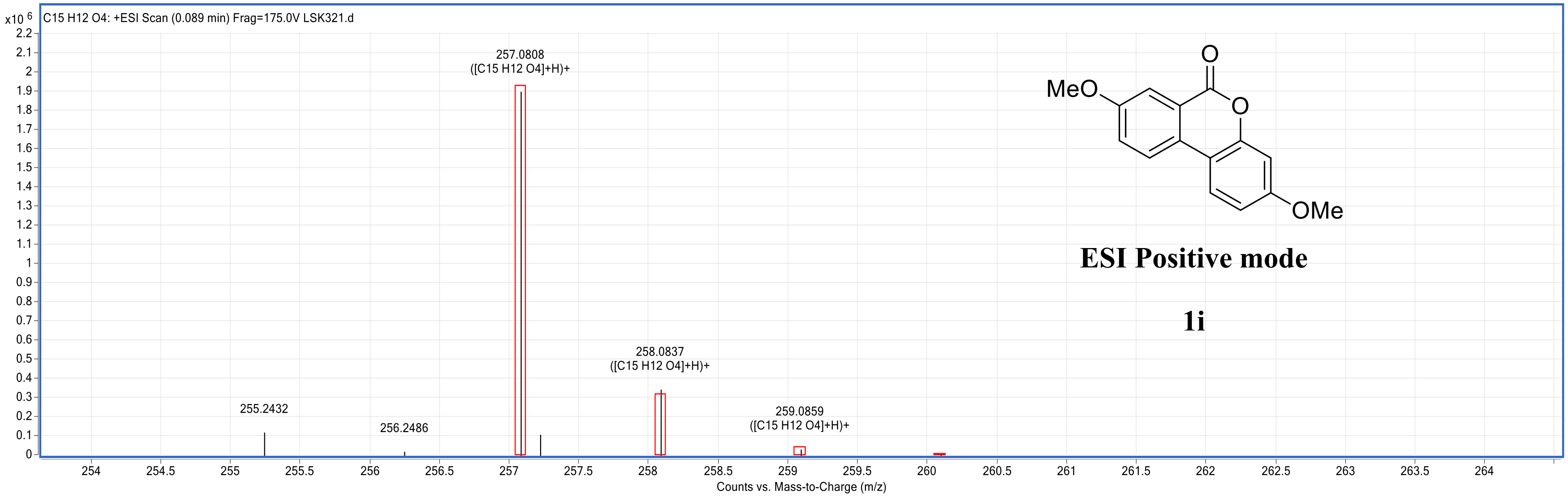
x10 6
C15 H12 O4: +ESI Scan (0.089 min) Frag=175.0V LSK321.d
257.0808
([C15 H12 O4]+H)+
258.0837
([C15 H12 O4]+H)+
259.0859
([C15 H12 O4]+H)+
255.2432
256.2486
MeO
O
O
OMe
ESI Positive mode
1i
Counts vs. Mass-to-Charge (m/z)

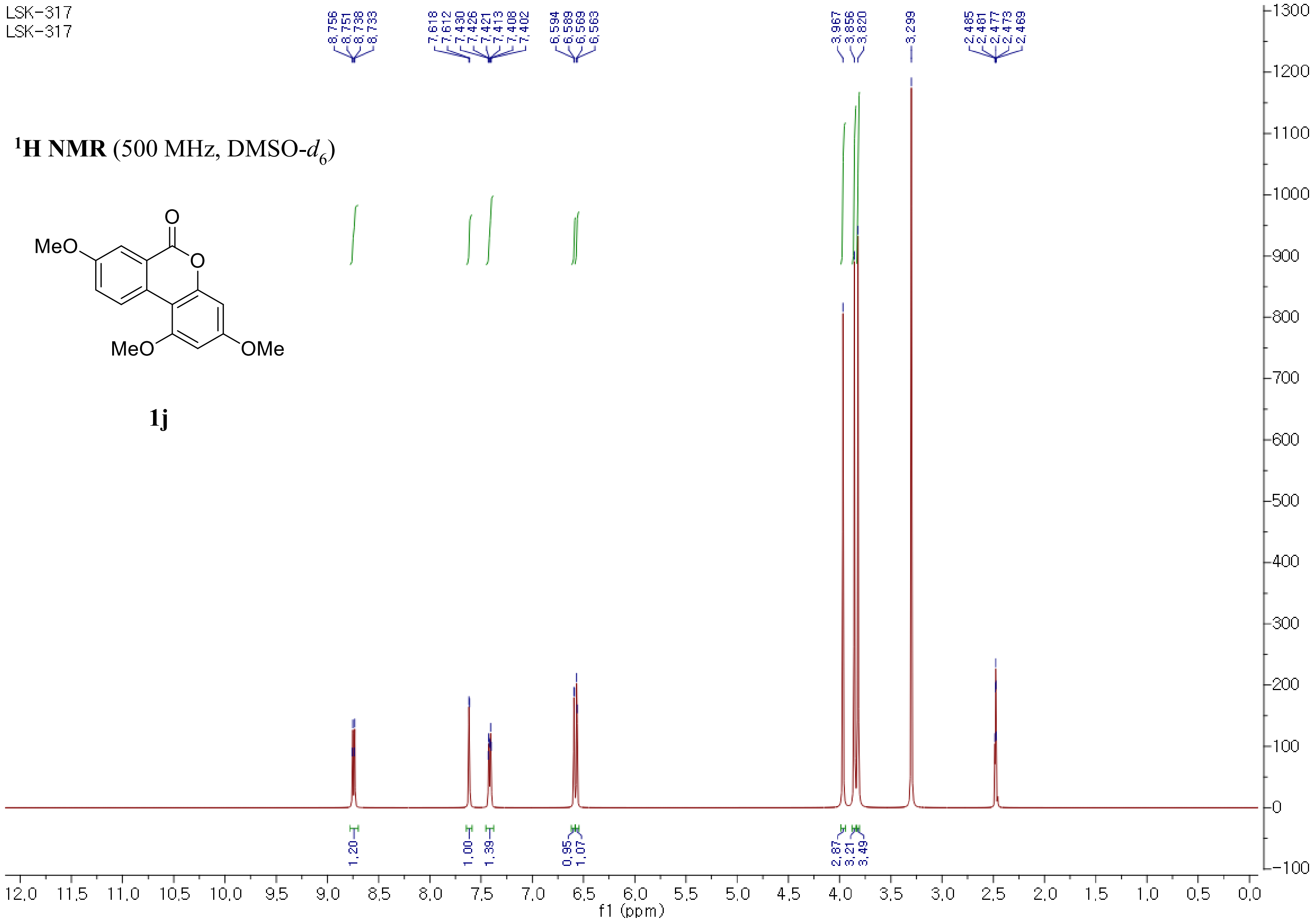
LSK-317
LSK-317
^{1}H NMR (500 MHz, DMSO-d_6)
MeO
O
O
MeO
OMe
1j
8.756 8.751 8.738 8.733
7.618 7.612 7.430 7.426 7.421 7.413 7.408 7.402
6.594 6.589 6.569 6.563
3.967 3.856 3.820
3.299
2.485 2.481 2.477 2.473 2.469
1.20
1.00 1.39
0.95 1.07
2.87 3.21 3.49
f1 (ppm)

^{13}C NMR (100 MHz, DMSO-d_6)

1j

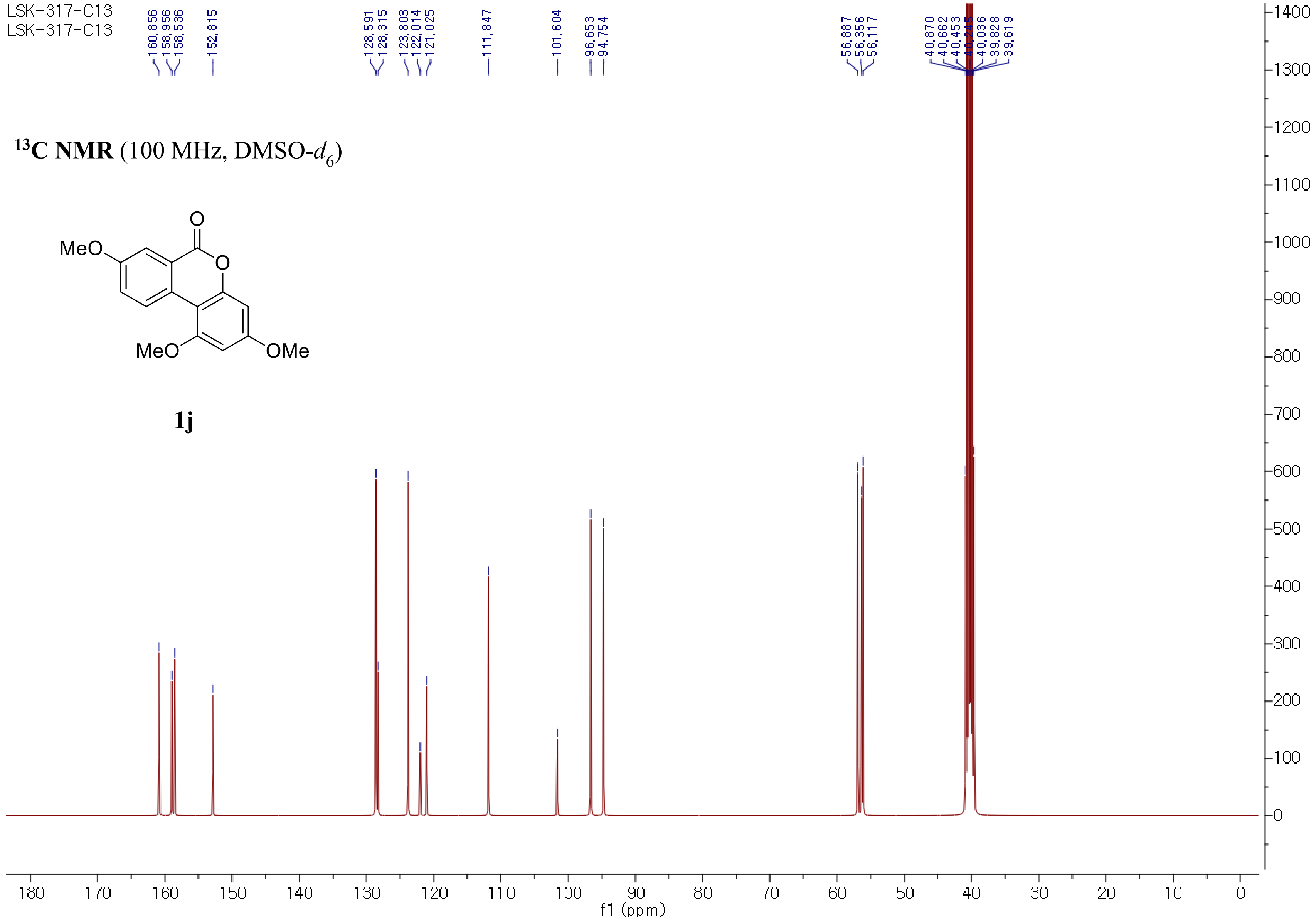

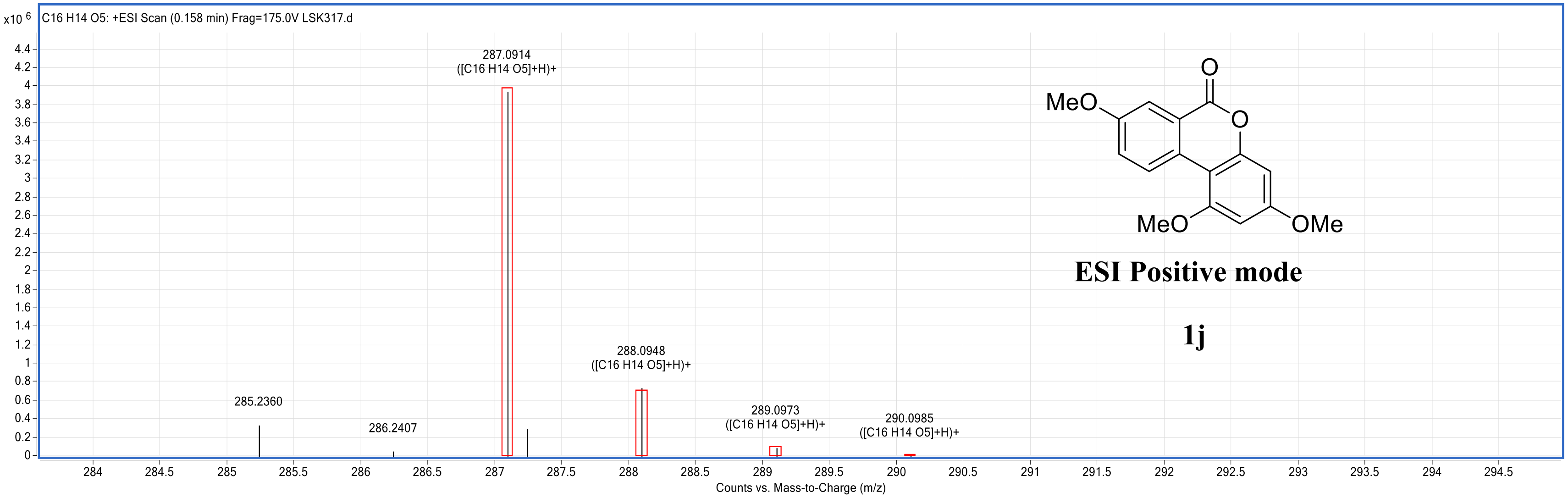
x10 6
C16 H14 O5: +ESI Scan (0.158 min) Frag=175.0V LSK317.d
287.0914
([C16 H14 O5]+H)+
288.0948
([C16 H14 O5]+H)+
289.0973
([C16 H14 O5]+H)+
290.0985
([C16 H14 O5]+H)+
285.2360
286.2407
Counts vs. Mass-to-Charge (m/z)
MeO
O
O
MeO
OMe
ESI Positive mode
1j

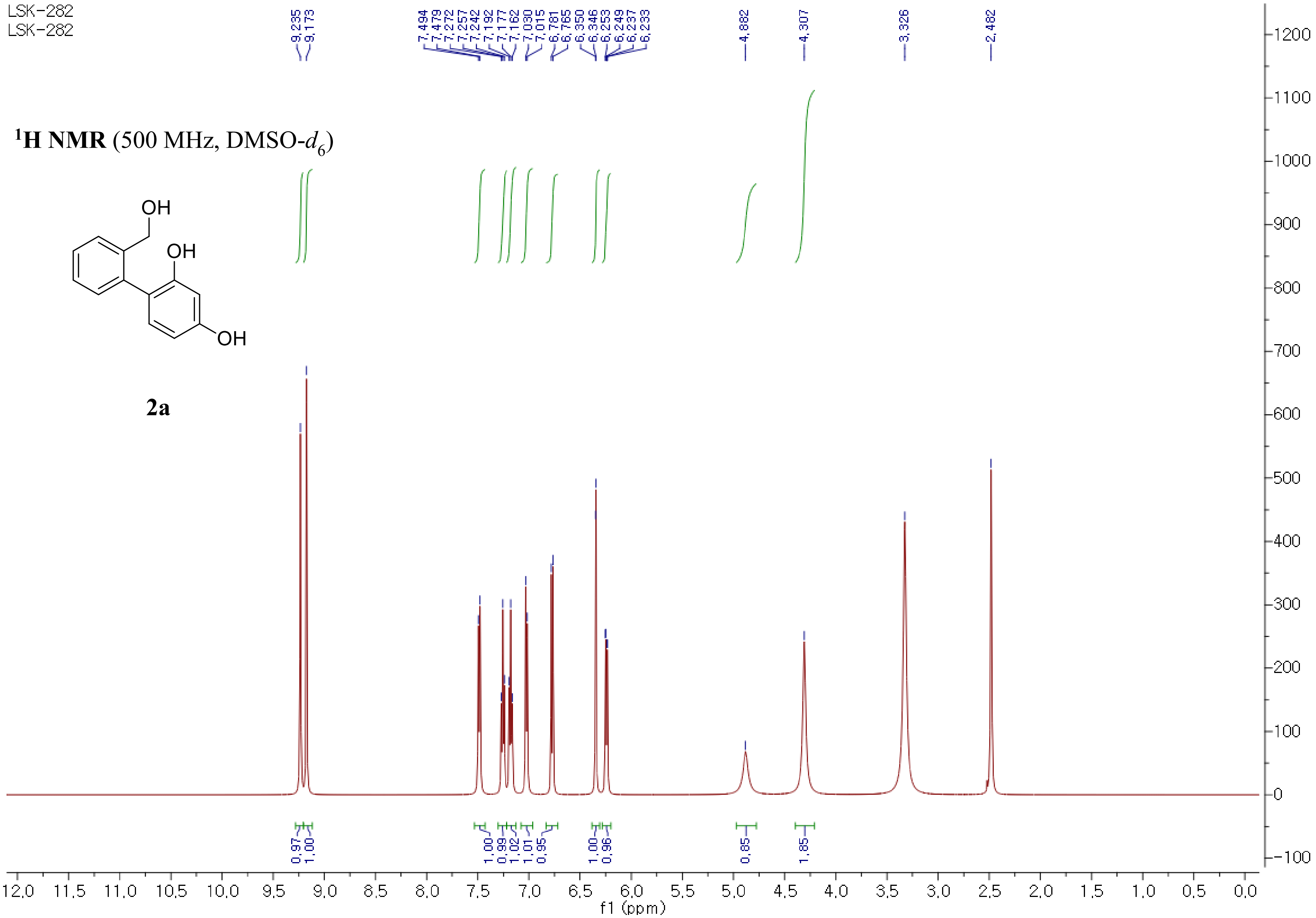
LSK-282
LSK-282
1H NMR (500 MHz, DMSO-d6)
OH
OH
OH
2a
9.235
9.173
7.494
7.479
7.272
7.257
7.242
7.192
7.177
7.162
7.030
7.015
6.781
6.765
6.350
6.346
6.253
6.249
6.237
6.233
4.882
4.307
3.326
2.482
0.97
1.00
1.00
0.99
1.02
1.01
0.95
1.00
0.96
0.85
1.85
f1 (ppm)

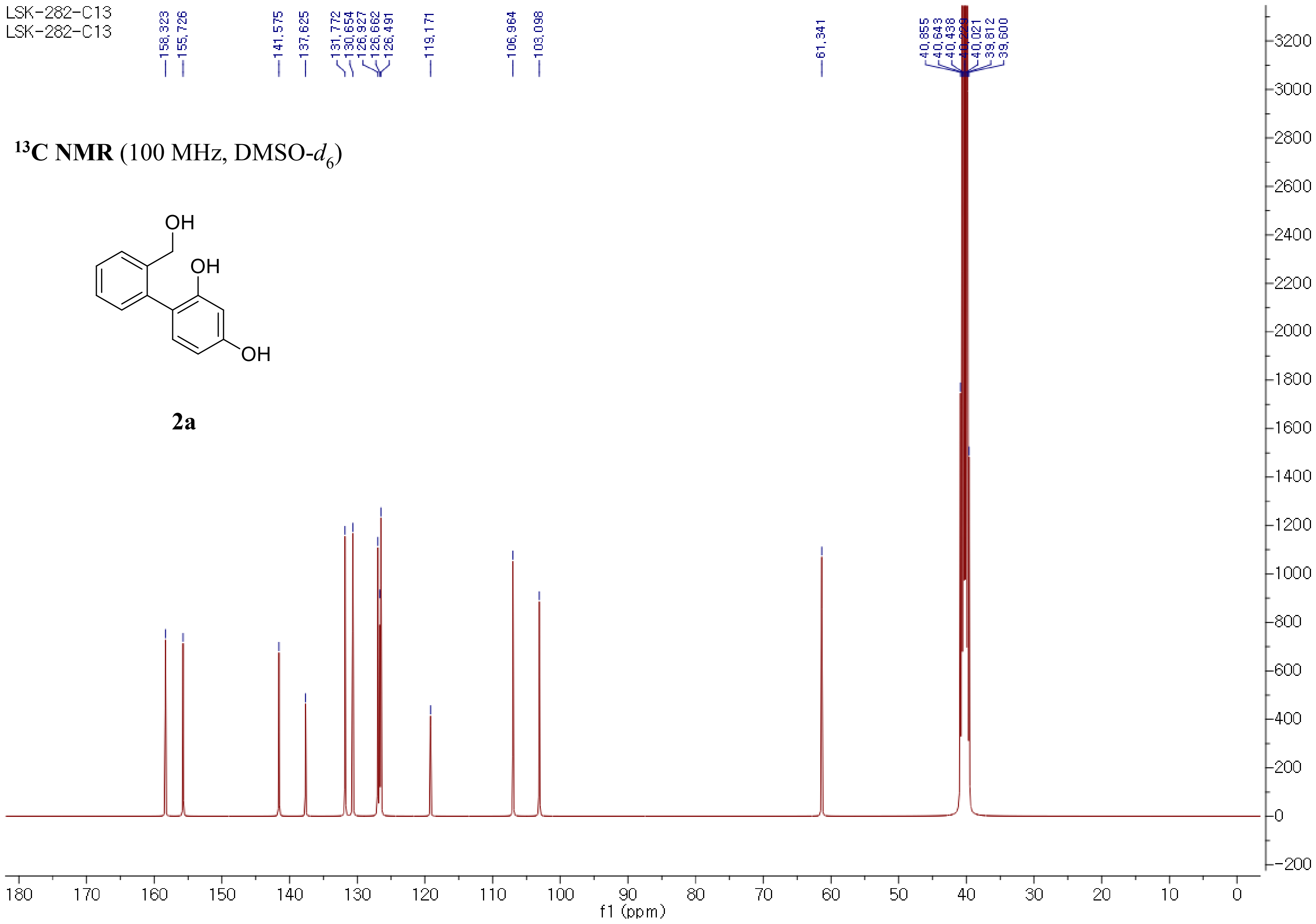
LSK-282-C13
LSK-282-C13
158.323
155.726
141.575
137.625
131.772
130.654
126.927
126.662
126.491
119.171
106.964
103.098
61.341
40.855
40.643
40.438
40.021
39.812
39.600
^{13}C NMR (100 MHz, DMSO-d_6)
OH
OH
OH
2a
f1 (ppm)

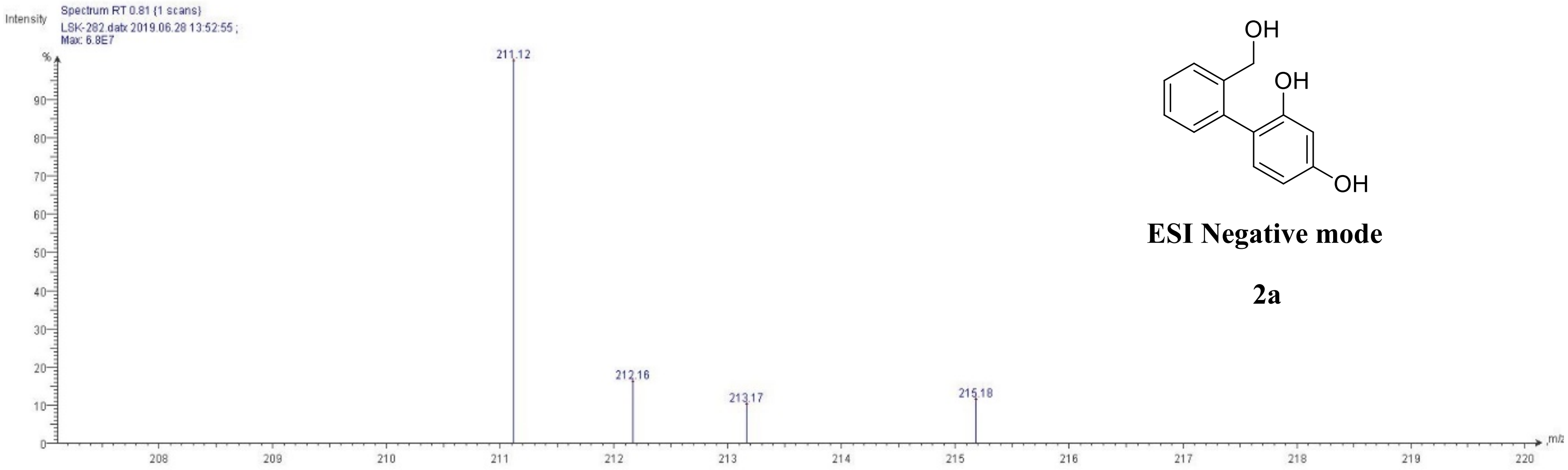

ESI Negative mode

2a

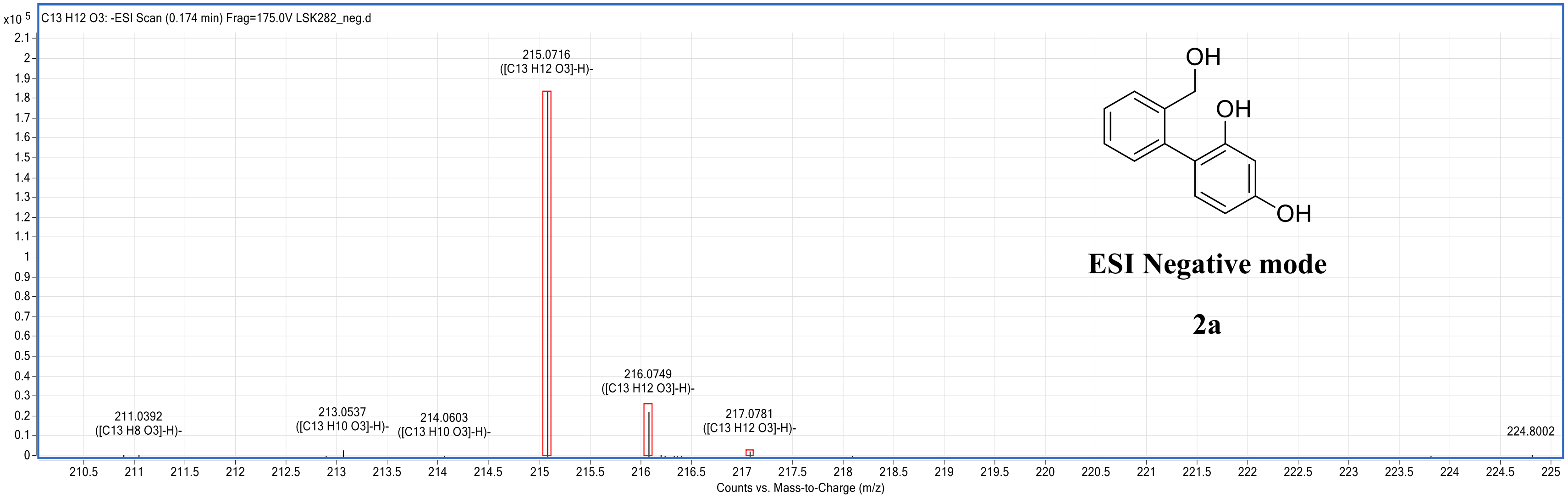
x10 5
C13 H12 O3: -ESI Scan (0.174 min) Frag=175.0V LSK282_neg.d
215.0716
([C13 H12 O3]-H)-
216.0749
([C13 H12 O3]-H)-
217.0781
([C13 H12 O3]-H)-
211.0392
([C13 H8 O3]-H)-
213.0537
([C13 H10 O3]-H)-
214.0603
([C13 H10 O3]-H)-
224.8002
Counts vs. Mass-to-Charge (m/z)
OH
OH
OH
ESI Negative mode
2a

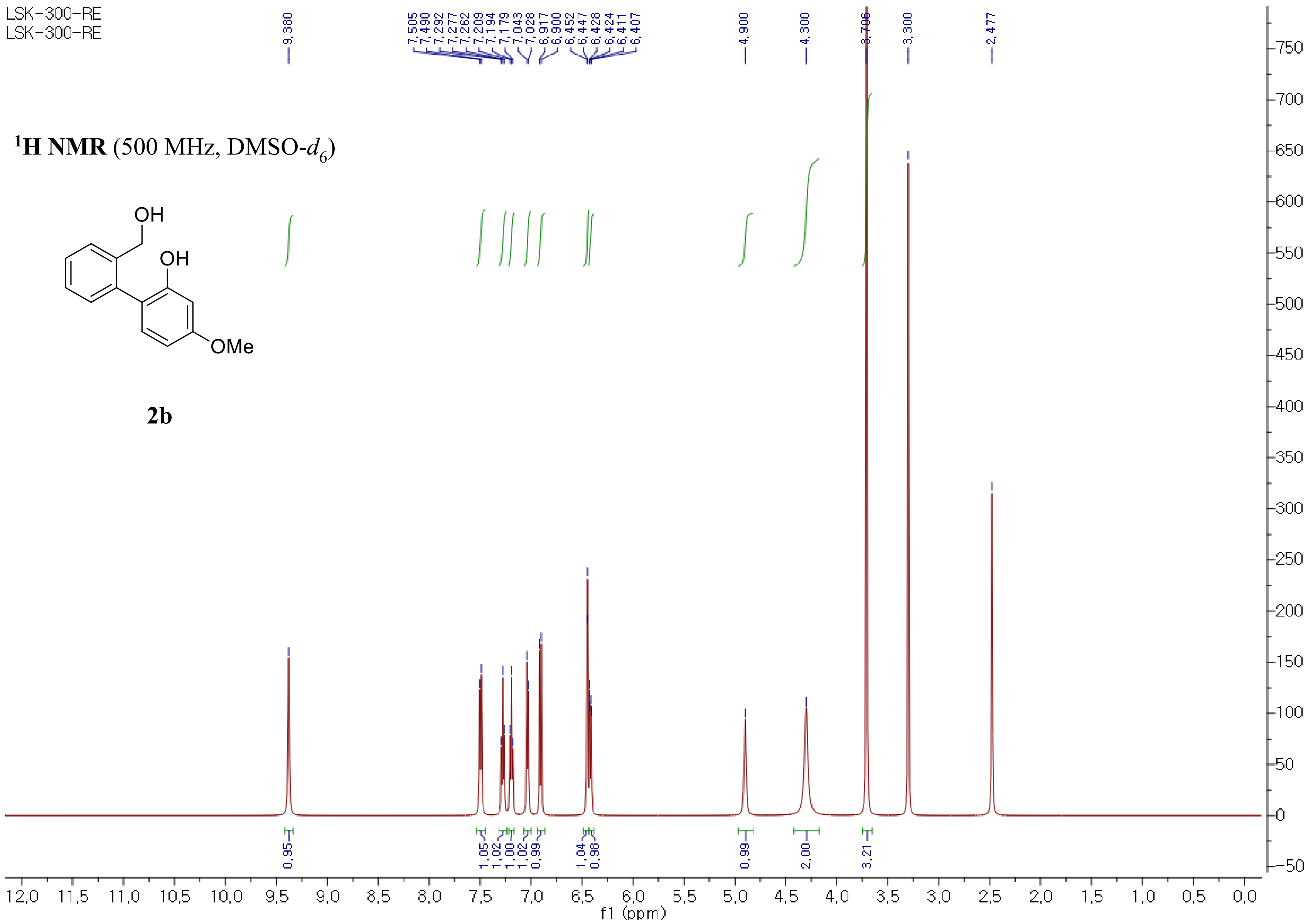
LSK-300-RE
LSK-300-RE
9.380
7.505 7.490 7.292 7.277 7.262 7.209 7.194 7.179 7.043 7.028 6.917 6.900 6.452 6.447 6.428 6.424 6.411 6.407
4.900
4.300
3.706
3.300
2.477
1H NMR (500 MHz, DMSO-d6)
OH
OH
OMe
2b
0.95
1.05 1.02 1.00 1.02 0.99
1.04 0.98
0.99
2.00
3.21
f1 (ppm)

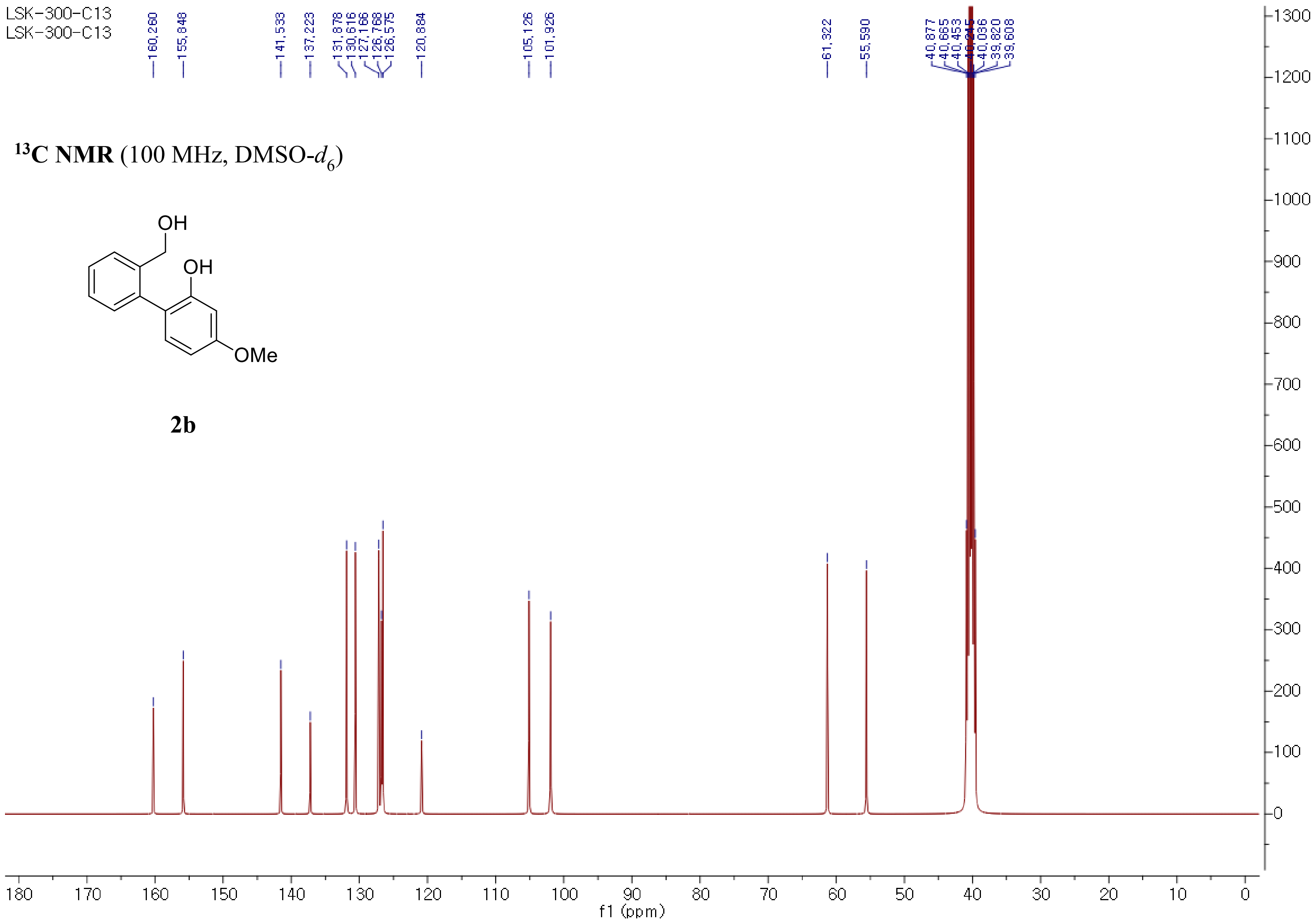
LSK-300-C13
LSK-300-C13
160.260
155.848
141.533
137.223
131.878
130.616
127.166
126.768
126.575
120.884
105.126
101.926
61.322
55.590
40.877
40.665
40.453
40.036
39.820
39.608
^{13}C NMR (100 MHz, DMSO-d_6)
OH
OH
OMe
2b
1300
1200
1100
1000
900
800
700
600
500
400
300
200
100
0
180
170
160
150
140
130
120
110
100
90
80
70
60
50
40
30
20
10
0
f1 (ppm)

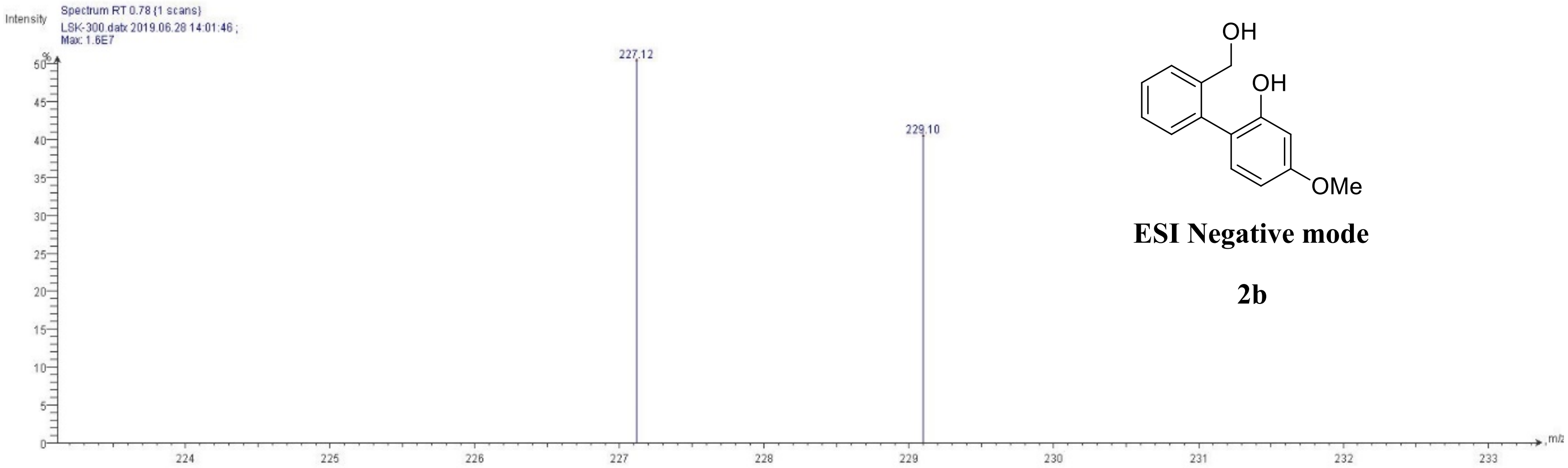

ESI Negative mode

2b

^{1}**H NMR** (500 MHz, DMSO-d_6)

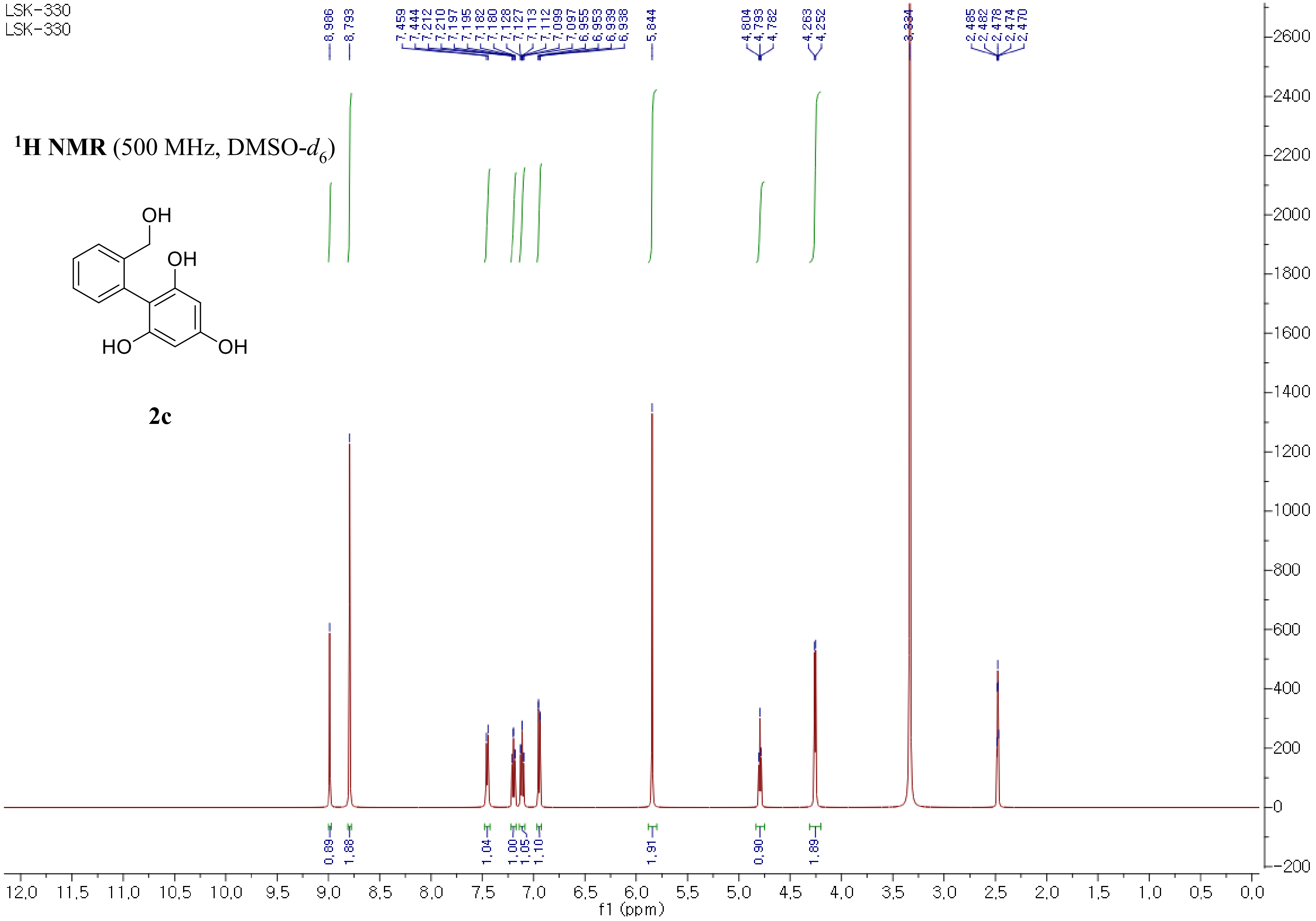

2c

^{13}C NMR (100 MHz, DMSO-d_6)

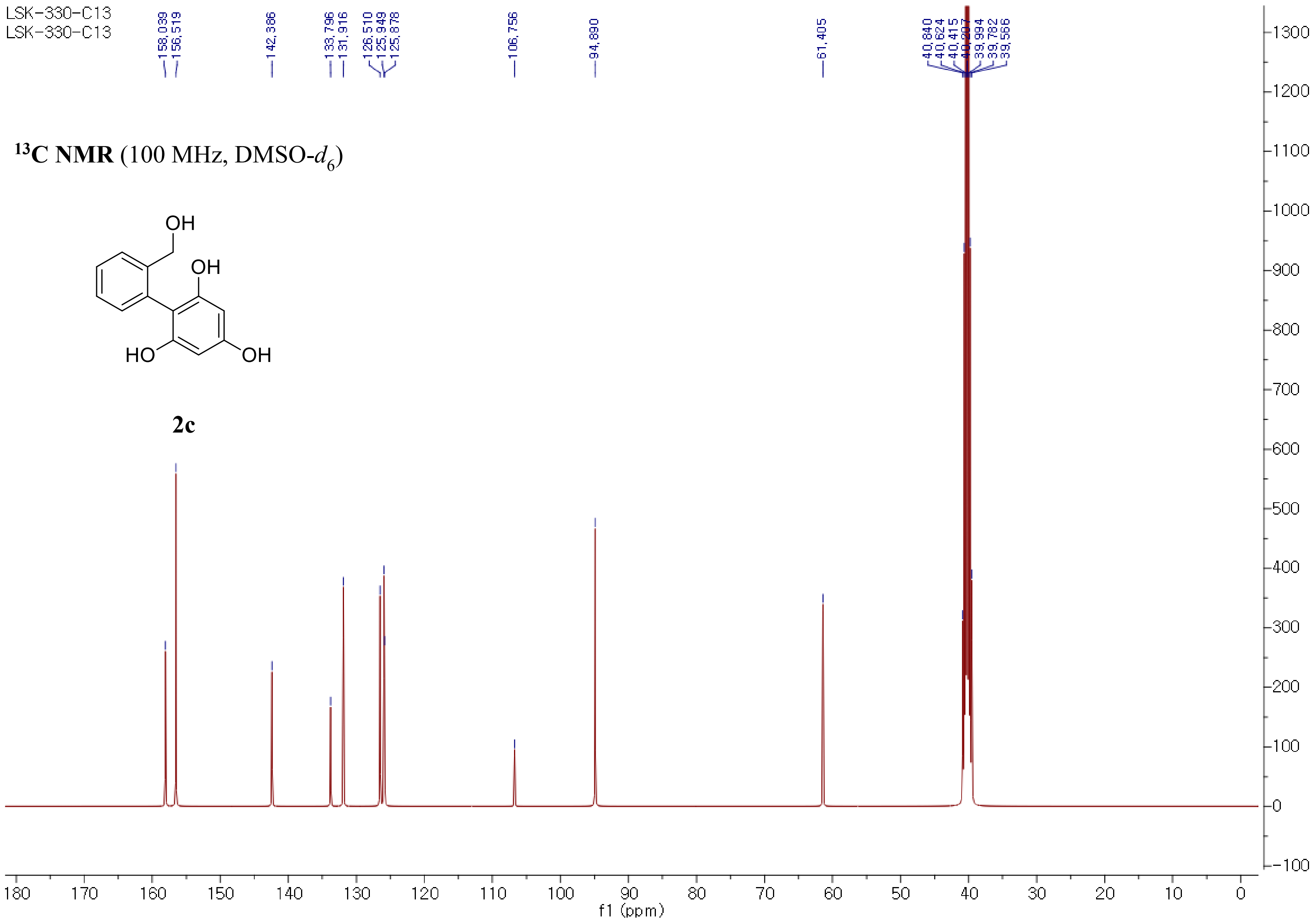

2c

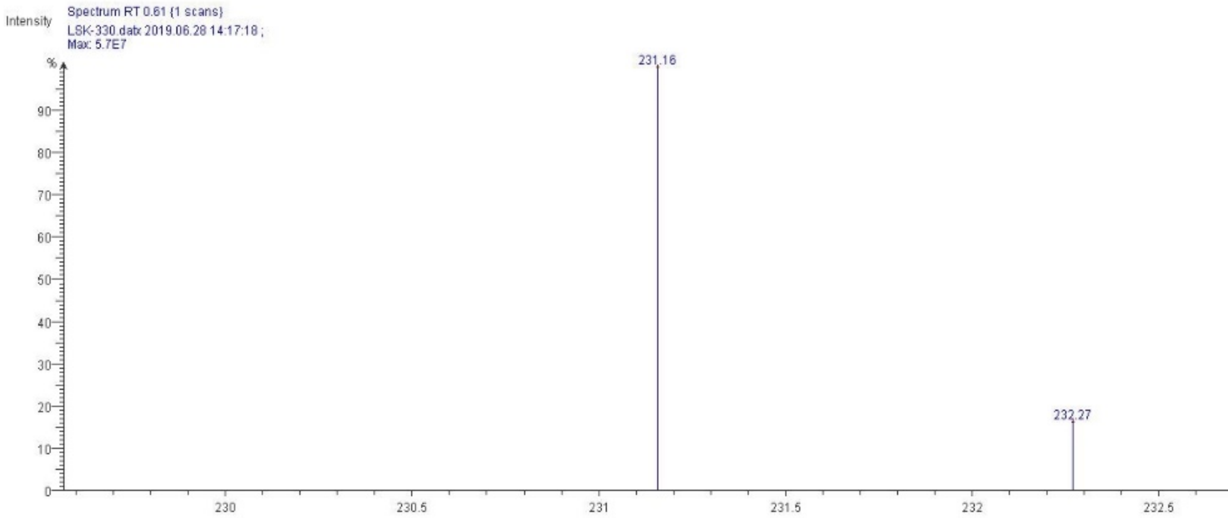

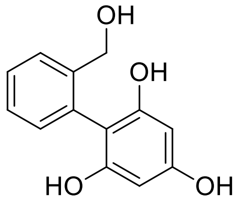

ESI Negative mode

2c

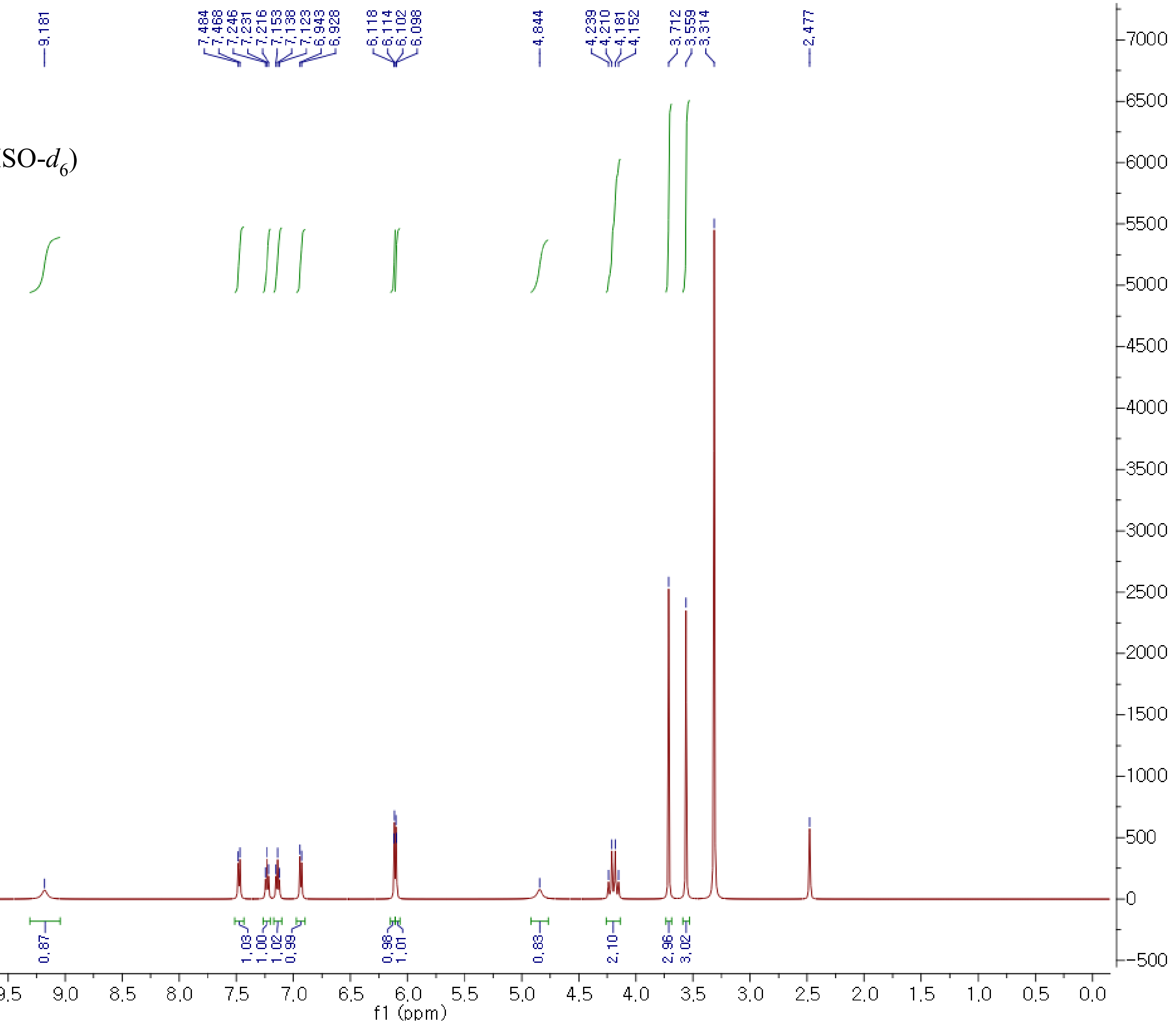

^{1}**H NMR** (500 MHz, DMSO-d_6)

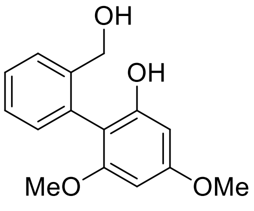

2d

^{13}C NMR (100 MHz, DMSO-d_6)

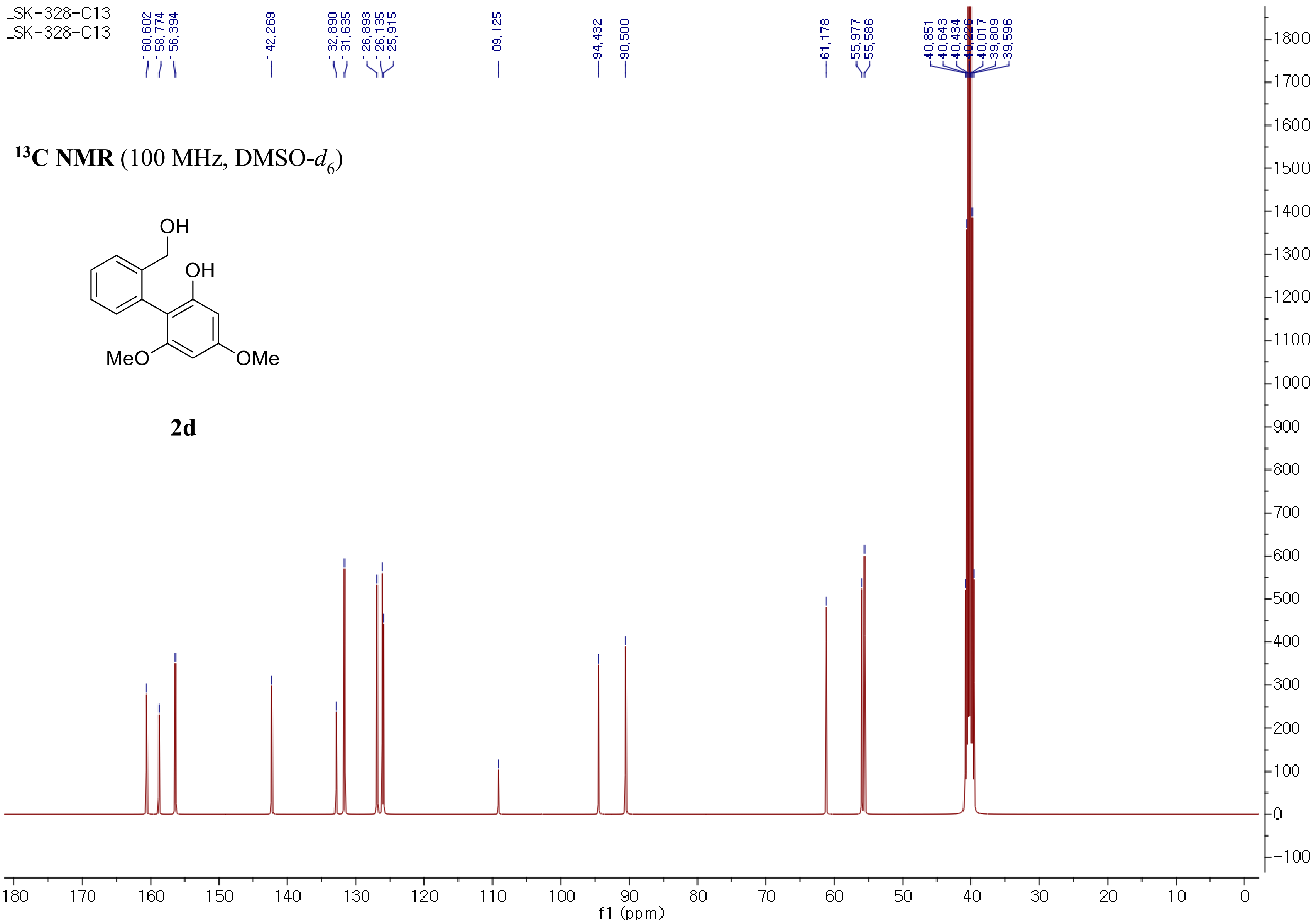

2d

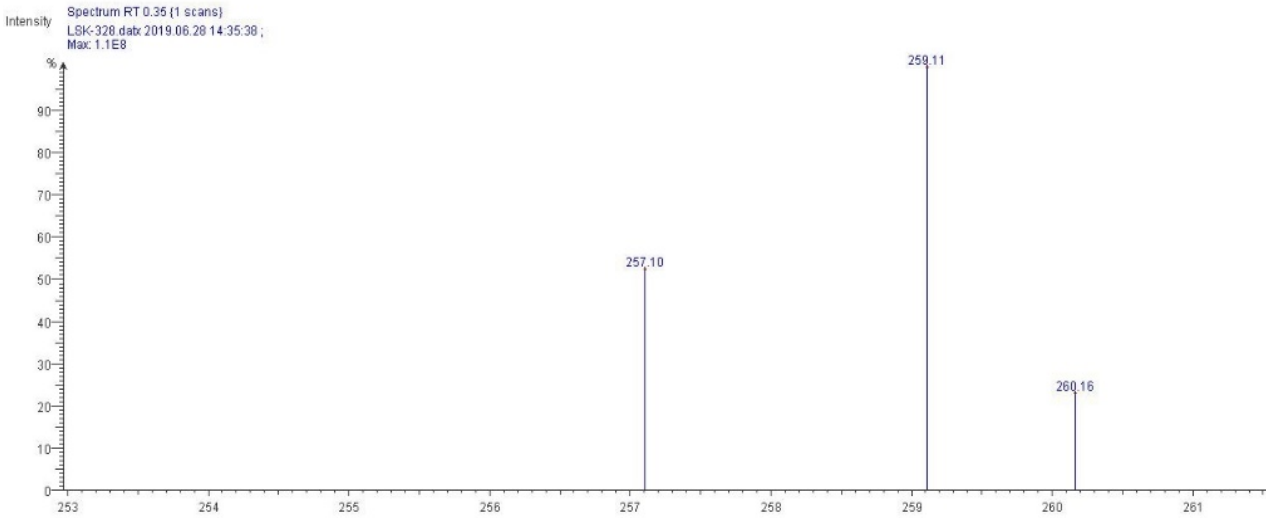

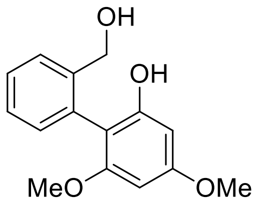

ESI Negative mode

2d

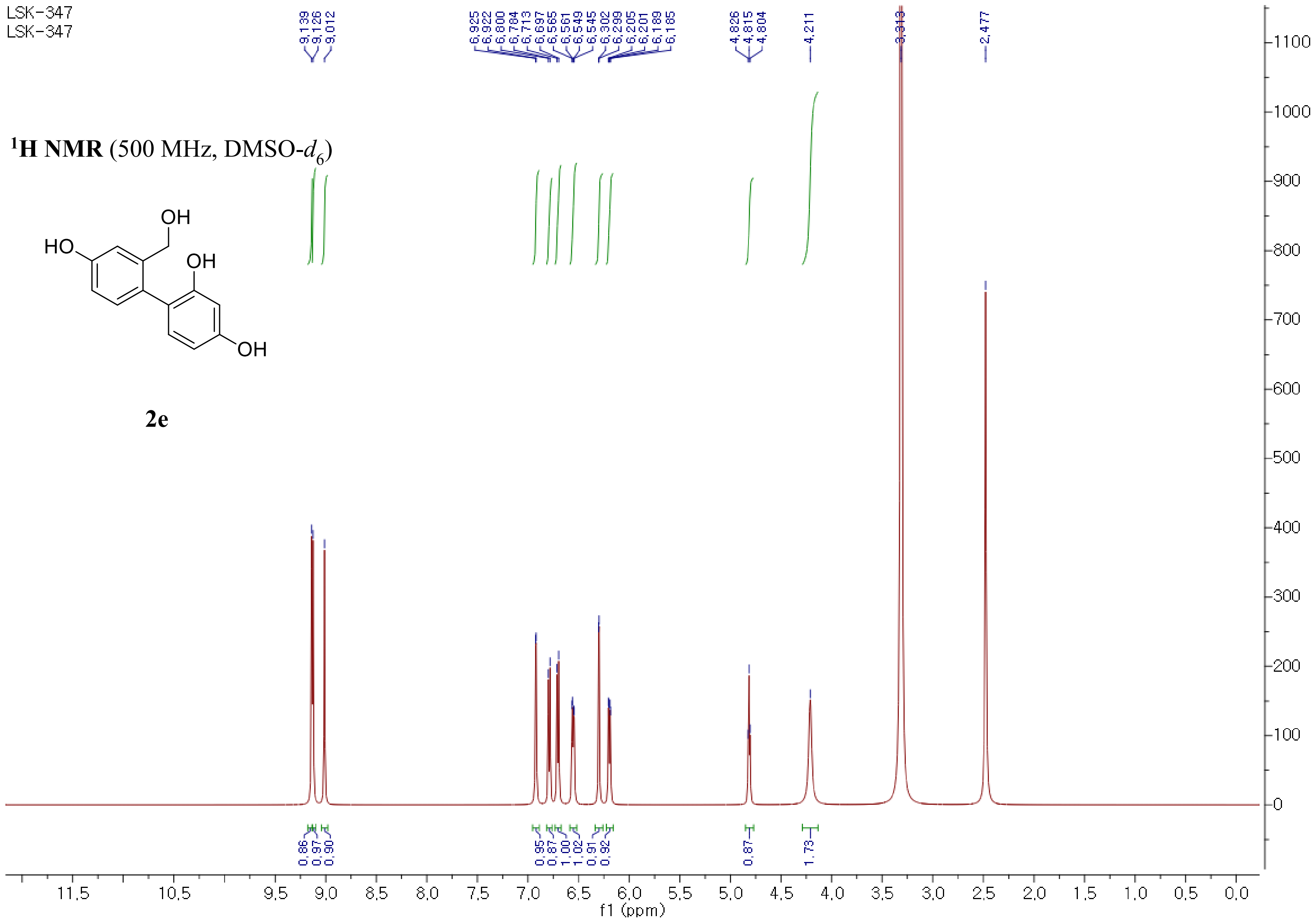
LSK-347
LSK-347
¹H NMR (500 MHz, DMSO-d6)
HO
OH
OH
OH
2e
9.139
9.126
9.012
6.925
6.922
6.800
6.784
6.713
6.697
6.565
6.561
6.549
6.545
6.302
6.299
6.205
6.201
6.189
6.185
4.826
4.815
4.804
4.211
3.313
2.477
0.86
0.97
0.90
0.95
0.87
1.00
1.02
0.91
0.92
0.87
1.73
f1 (ppm)

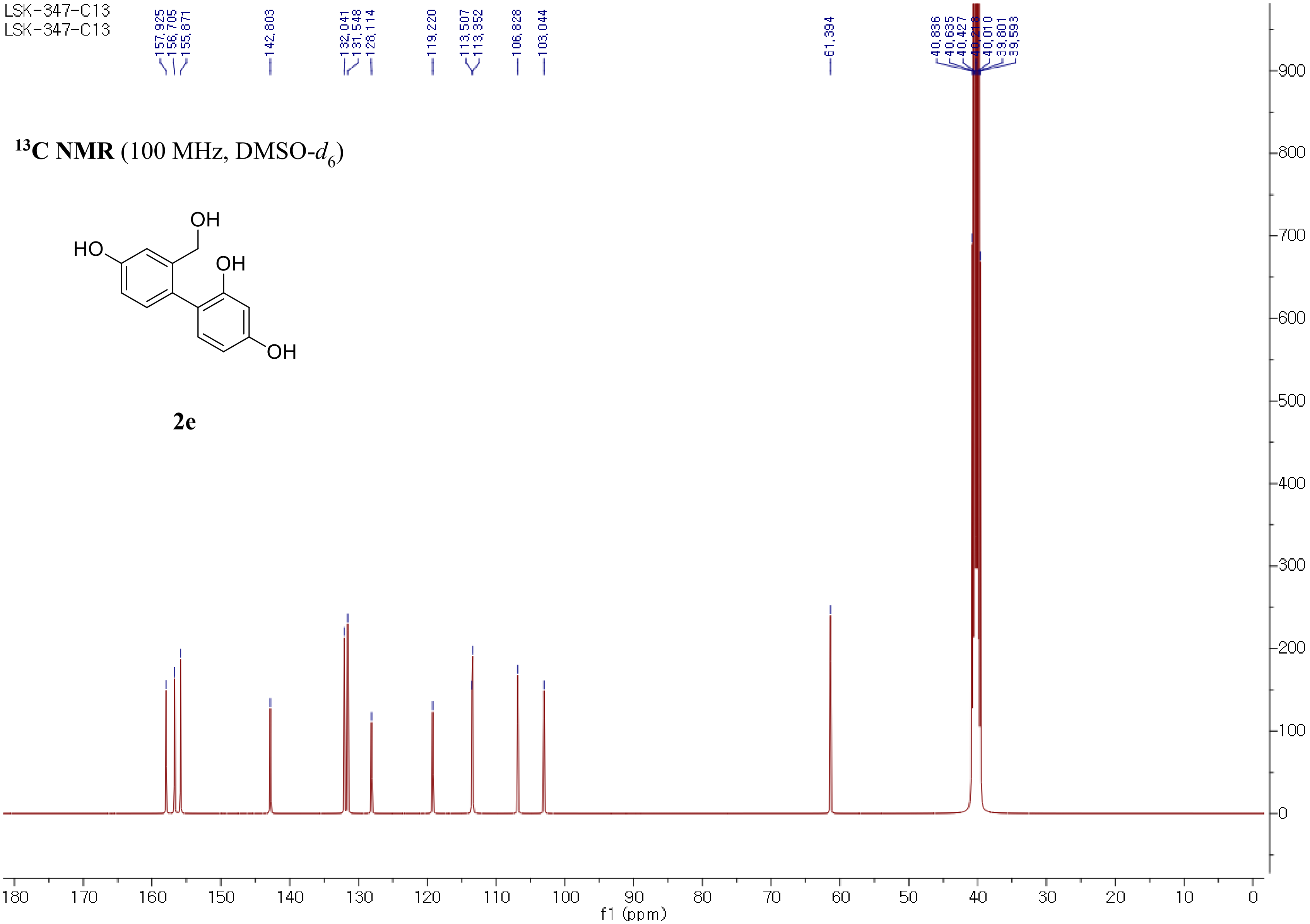

LSK-347-C13
LSK-347-C13
157.925
156.705
155.871
142.803
132.041
131.548
128.114
119.220
113.507
113.352
106.828
103.044
61.394
40.836
40.635
40.427
40.010
39.801
39.593
¹³C NMR (100 MHz, DMSO-d6)
OH
HO
OH
OH
2e
900
800
700
600
500
400
300
200
100
0
180 170 160 150 140 130 120 110 100 90 80 70 60 50 40 30 20 10 0
f1 (ppm)

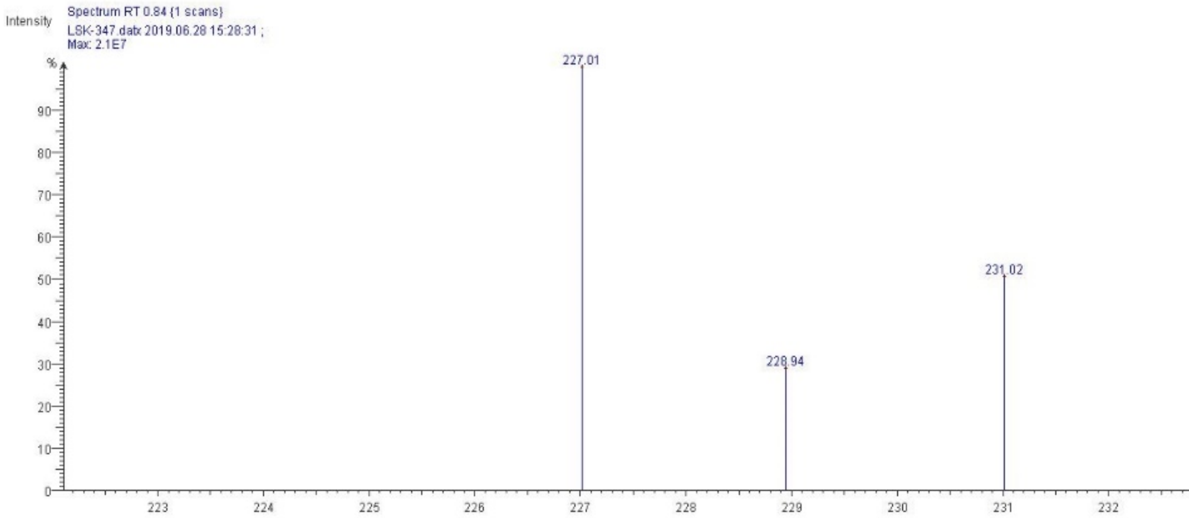

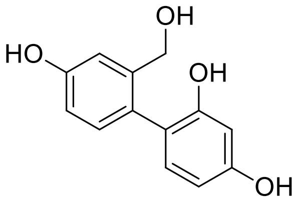

ESI Negative mode

2e

^{1}H NMR (500 MHz, DMSO-d_6)

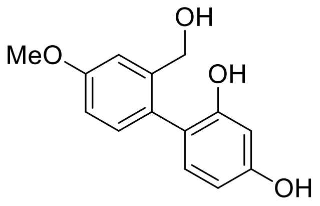

2f

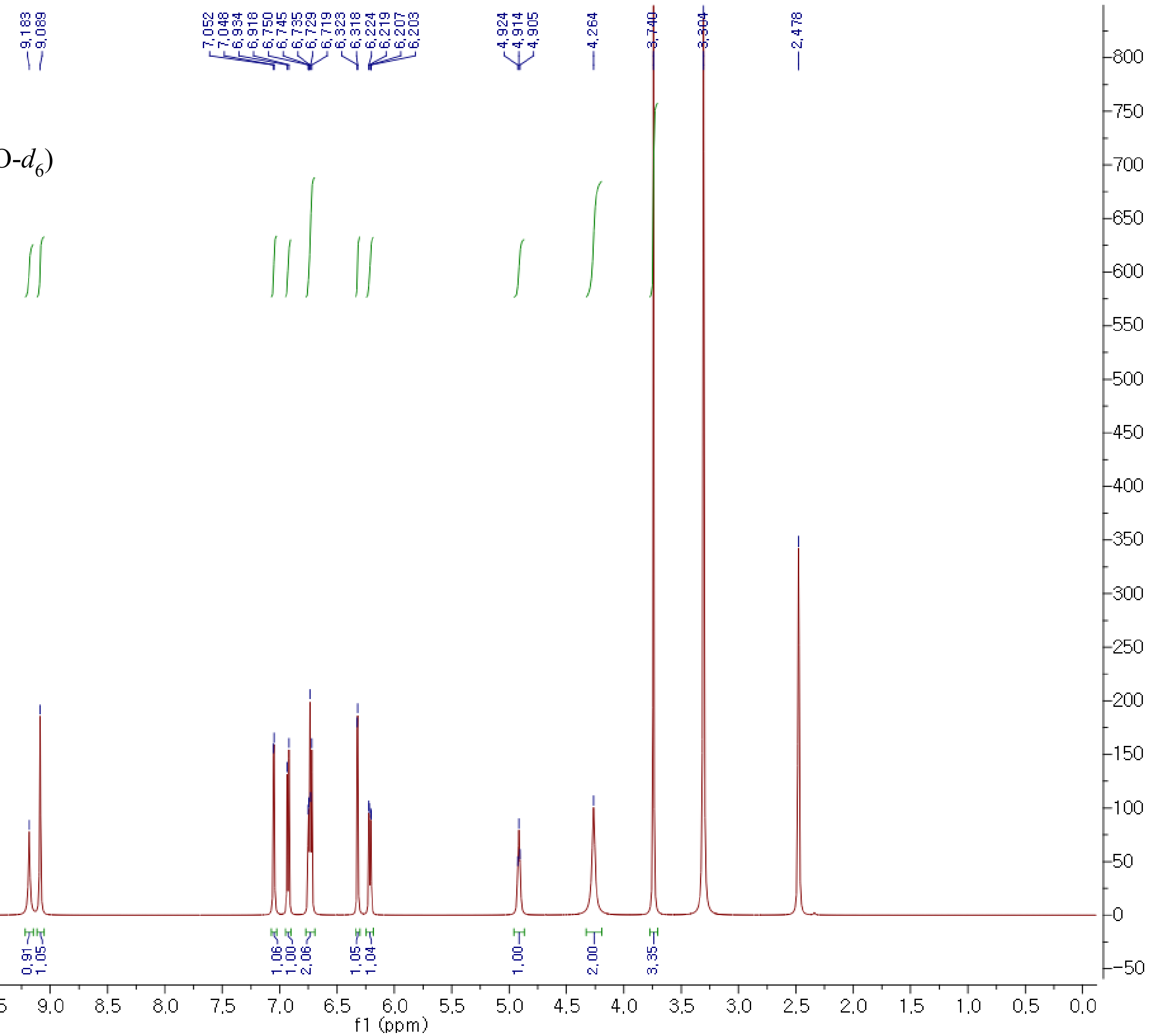

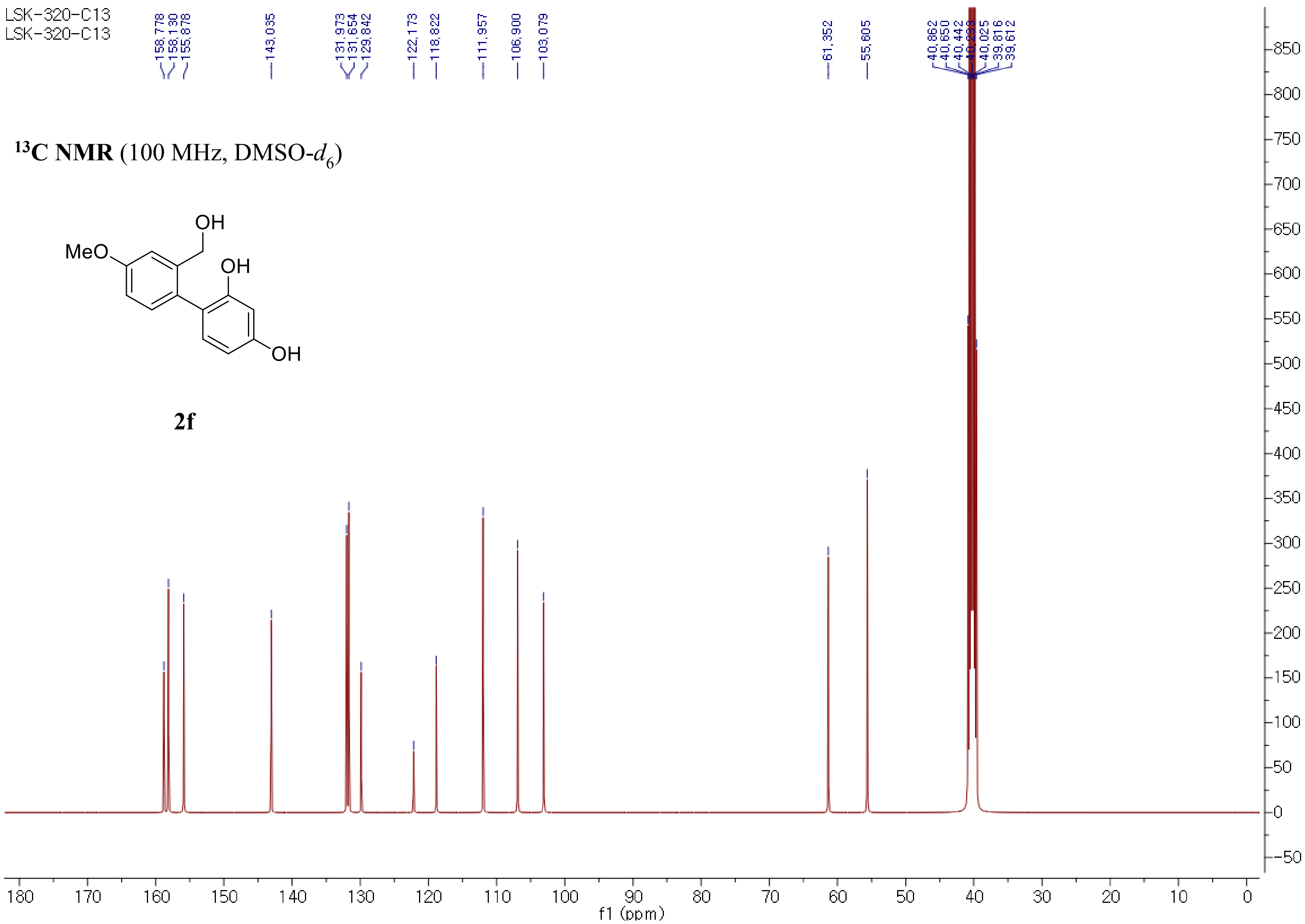
LSK-320-C13
LSK-320-C13
158.778
158.130
155.878
143.035
131.973
131.654
129.842
122.173
118.822
111.957
106.900
103.079
61.352
55.605
40.862
40.650
40.442
40.025
39.816
39.612
13C NMR (100 MHz, DMSO-d6)
MeO
OH
OH
OH
2f
f1 (ppm)

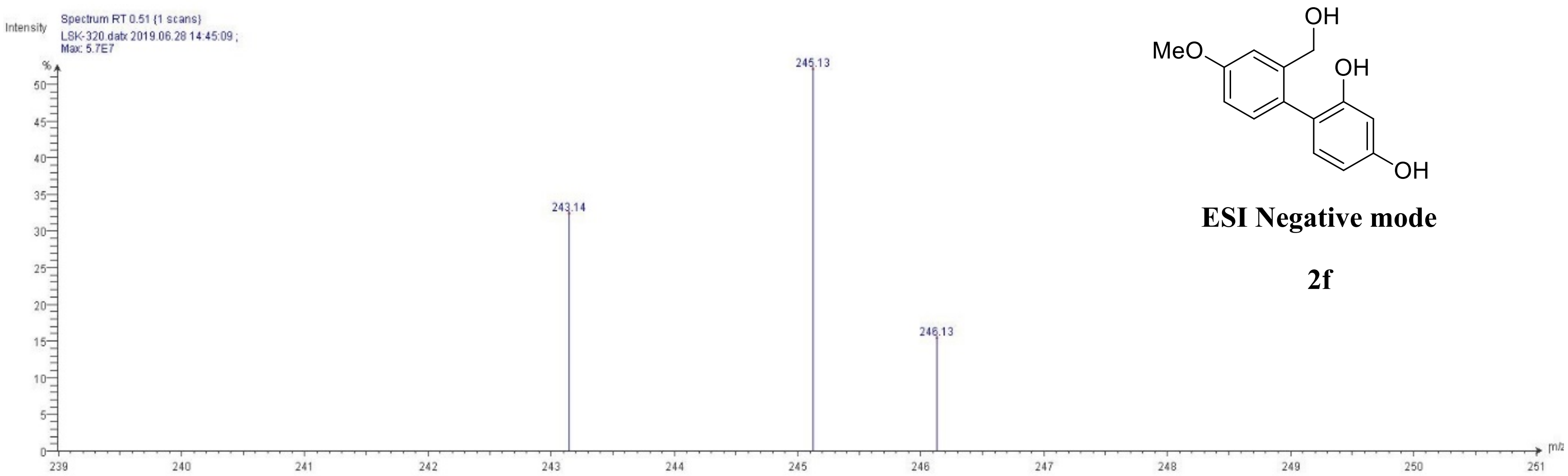

ESI Negative mode

2f

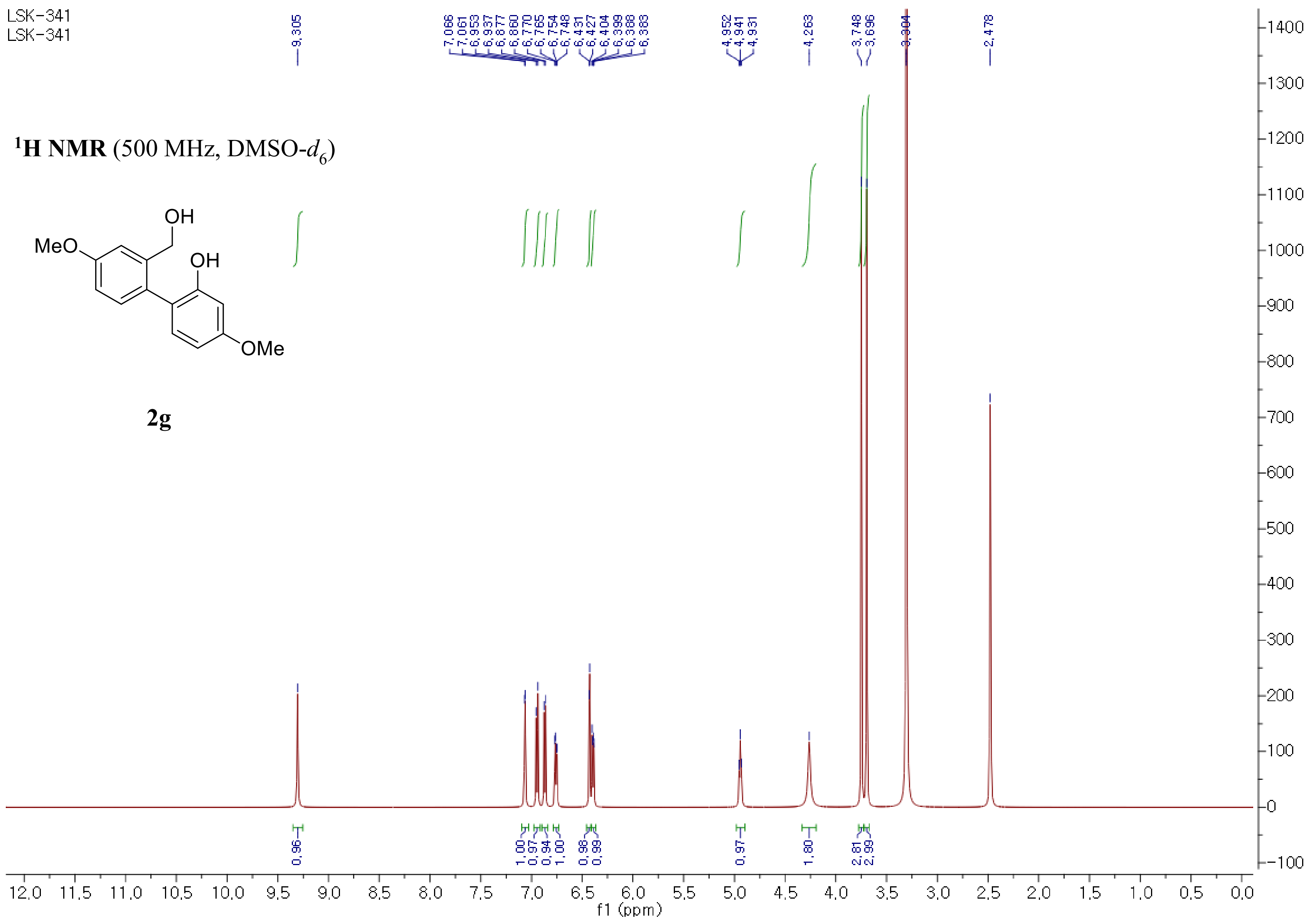
LSK-341
LSK-341
1H NMR (500 MHz, DMSO-d6)
OH
MeO
OH
OMe
2g
9.305
7.066 7.061 6.953 6.937 6.877 6.860 6.770 6.765 6.754 6.748 6.431 6.427 6.404 6.399 6.388 6.383
4.952 4.941 4.931
4.263
3.748 3.696
3.304
2.478
0.96
1.00 0.97 0.94 1.00
0.98 0.99
0.97
1.80
2.81 2.99
12.0 11.5 11.0 10.5 10.0 9.5 9.0 8.5 8.0 7.5 7.0 6.5 6.0 5.5 5.0 4.5 4.0 3.5 3.0 2.5 2.0 1.5 1.0 0.5 0.0
f1 (ppm)
1400 1300 1200 1100 1000 900 800 700 600 500 400 300 200 100 0 -100

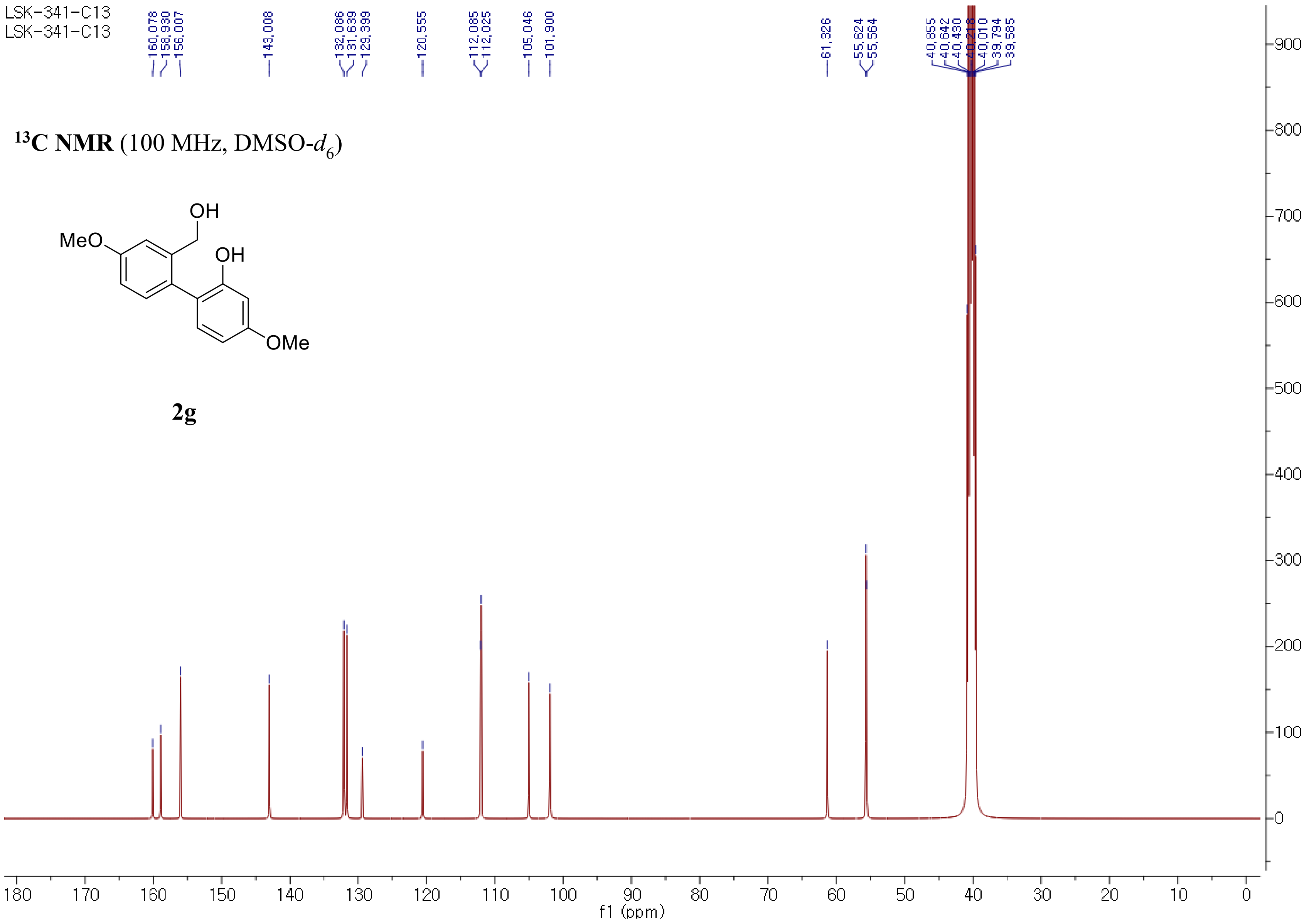

^{13}C NMR (100 MHz, DMSO-d_6)

2g

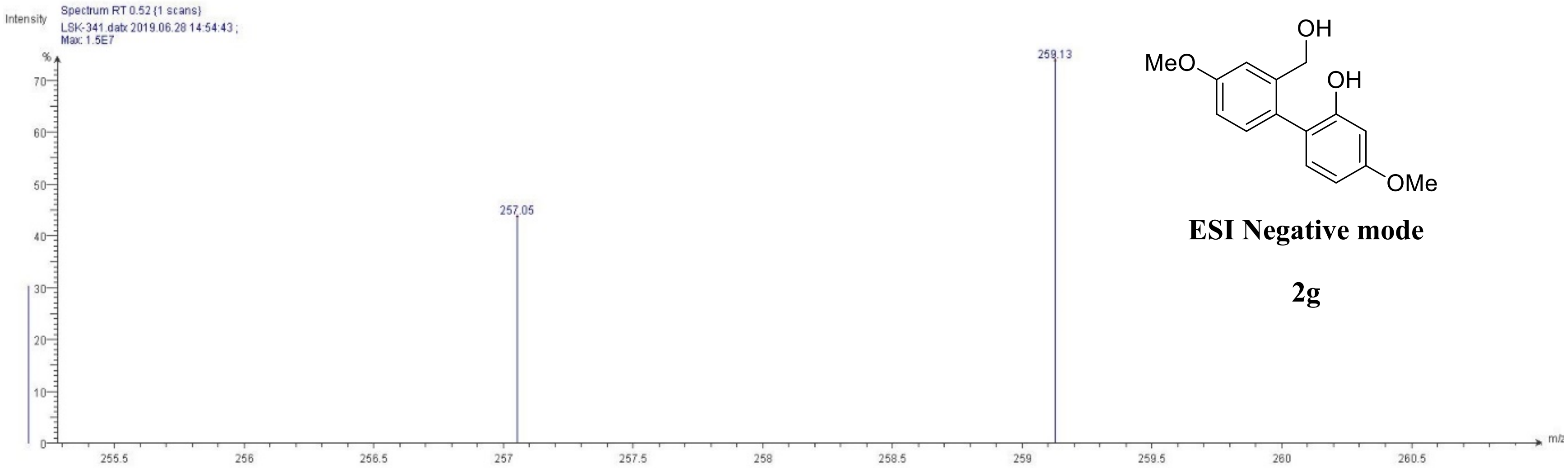

ESI Negative mode

2g

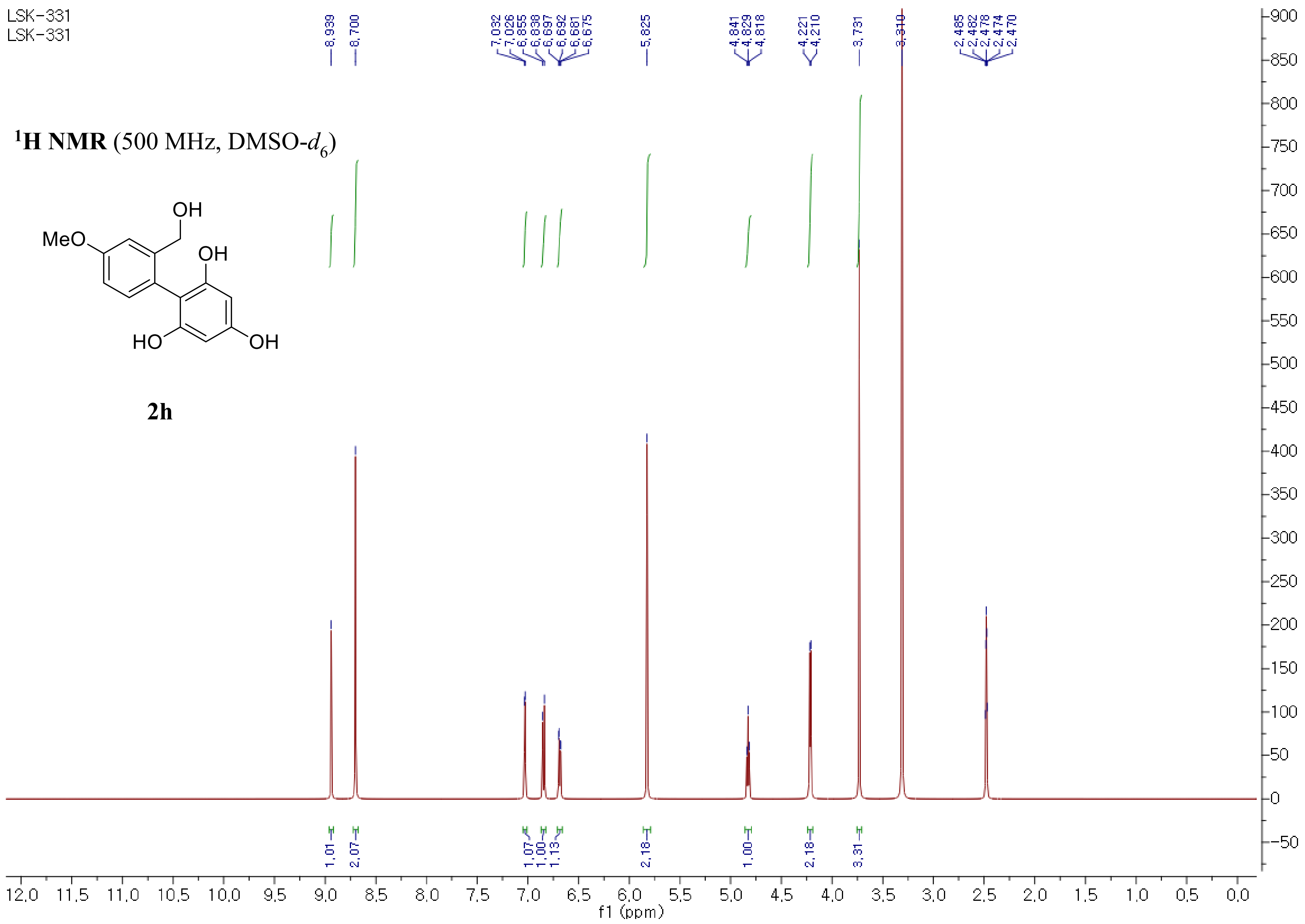
LSK-331
LSK-331
^{1}H NMR (500 MHz, DMSO-d_6)
MeO
OH
OH
HO
OH
2h
8.939
8.700
7.032
7.026
6.855
6.838
6.697
6.692
6.681
6.675
5.825
4.841
4.829
4.818
4.221
4.210
3.731
3.310
2.485
2.482
2.478
2.474
2.470
1.01
2.07
1.07
1.00
1.13
2.18
1.00
2.18
3.31
f1 (ppm)

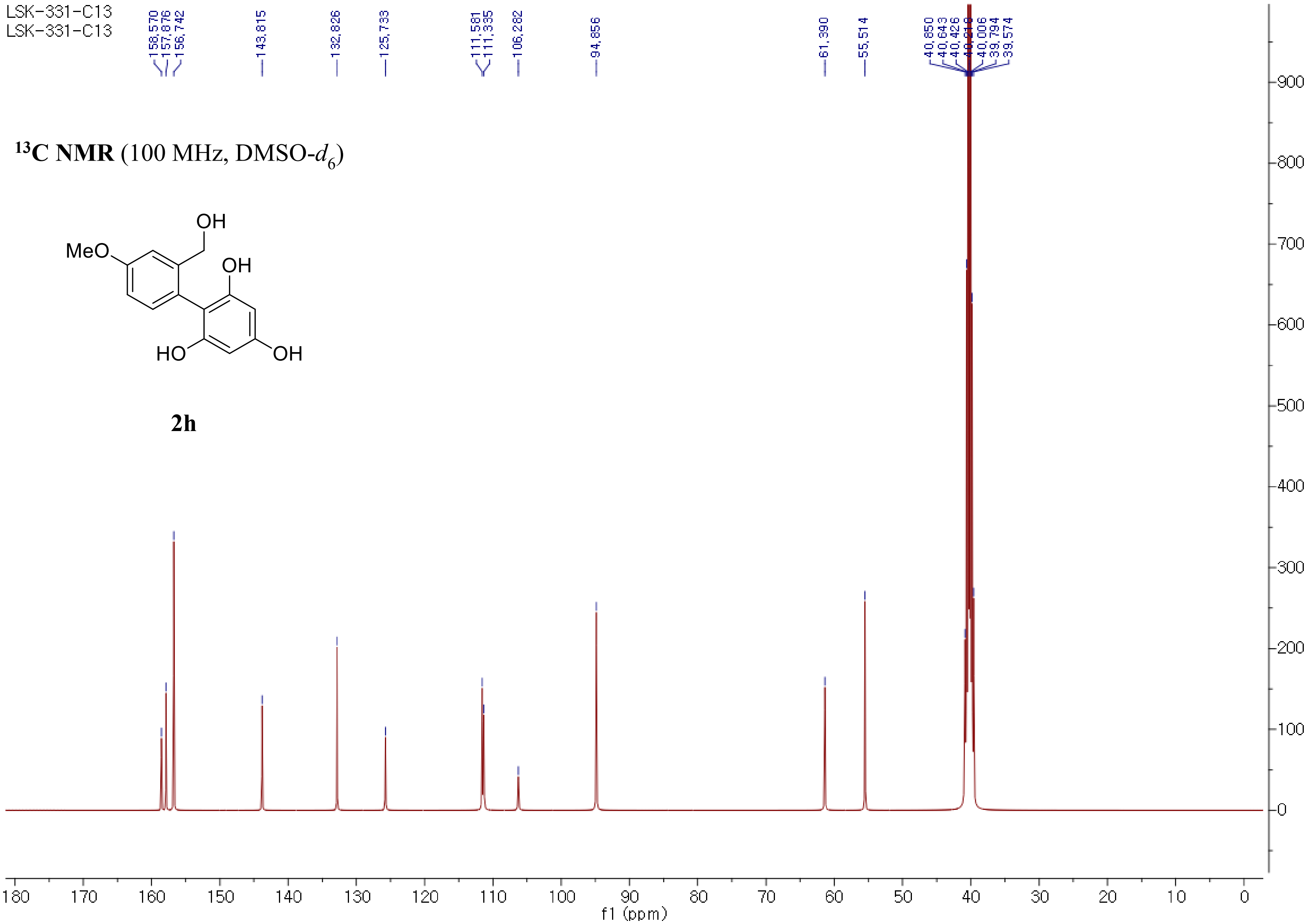
LSK-331-C13
LSK-331-C13
158.570
157.876
156.742
143.815
132.826
125.733
111.581
111.335
106.282
94.856
61.390
55.514
40.850
40.643
40.426
40.006
39.794
39.574
^{13}C NMR (100 MHz, DMSO-d_6)
MeO
OH
OH
HO
OH
2h
f1 (ppm)

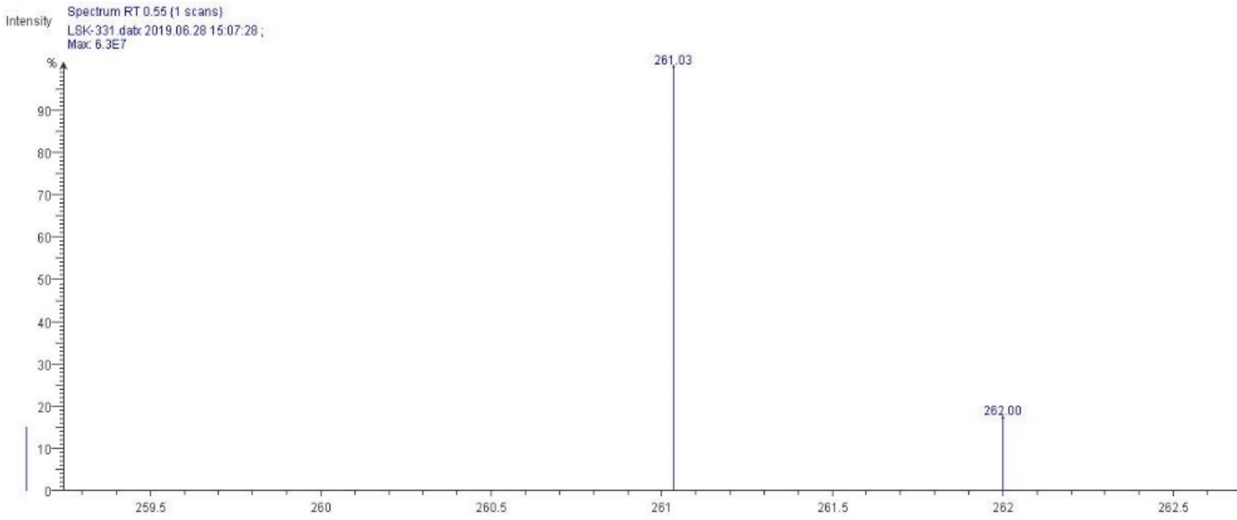

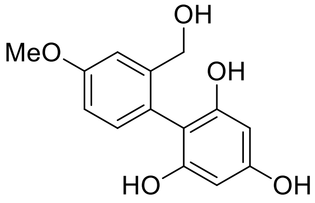

ESI Negative mode

2h

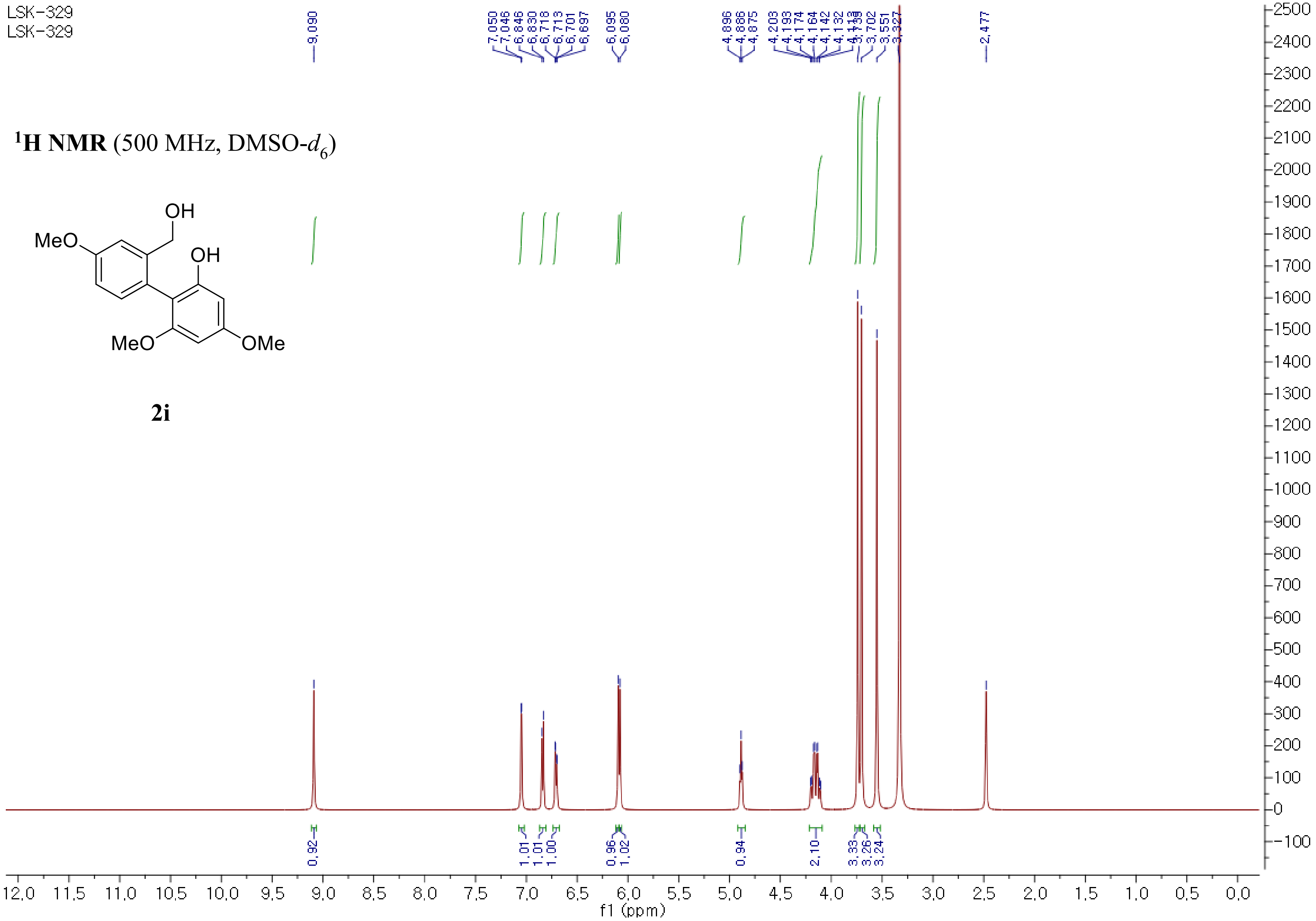

LSK-329
LSK-329
1H NMR (500 MHz, DMSO-d6)
OH
MeO
OH
MeO
OMe
2i
9.090
7.050
7.046
6.846
6.830
6.718
6.713
6.701
6.697
6.095
6.080
4.896
4.886
4.875
4.203
4.193
4.174
4.164
4.142
4.132
3.702
3.551
3.327
2.477
0.92
1.01
1.01
1.00
0.96
1.02
0.94
2.10
3.33
3.26
3.24
f1 (ppm)

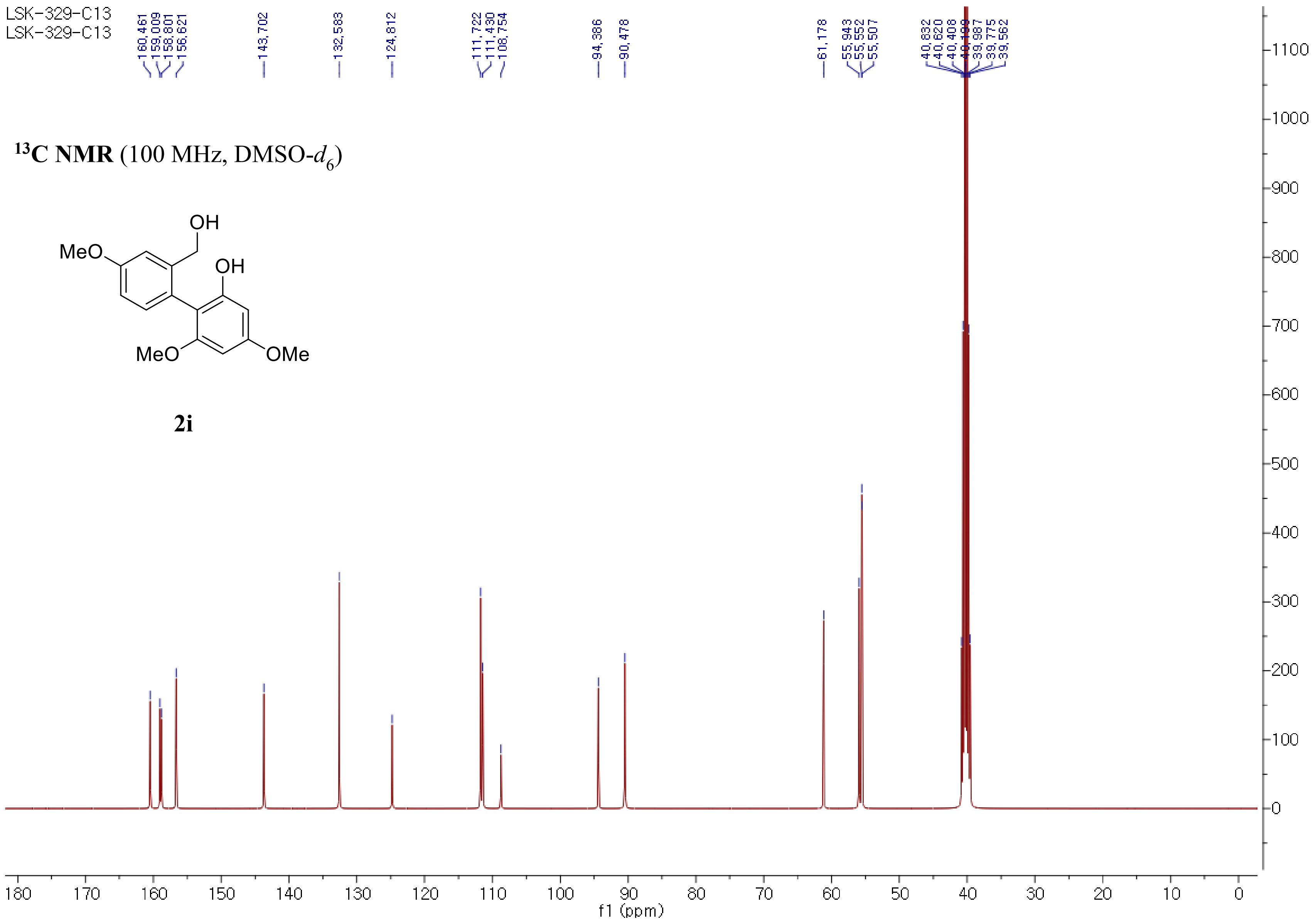
LSK-329-C13
LSK-329-C13
160.461
159.009
158.801
156.621
143.702
132.583
124.812
111.722
111.430
108.754
94.386
90.478
61.178
55.943
55.552
55.507
40.832
40.620
40.408
40.199
39.987
39.775
39.562
¹³C NMR (100 MHz, DMSO-d6)
OH
MeO
OH
MeO
OMe
2i
f1 (ppm)

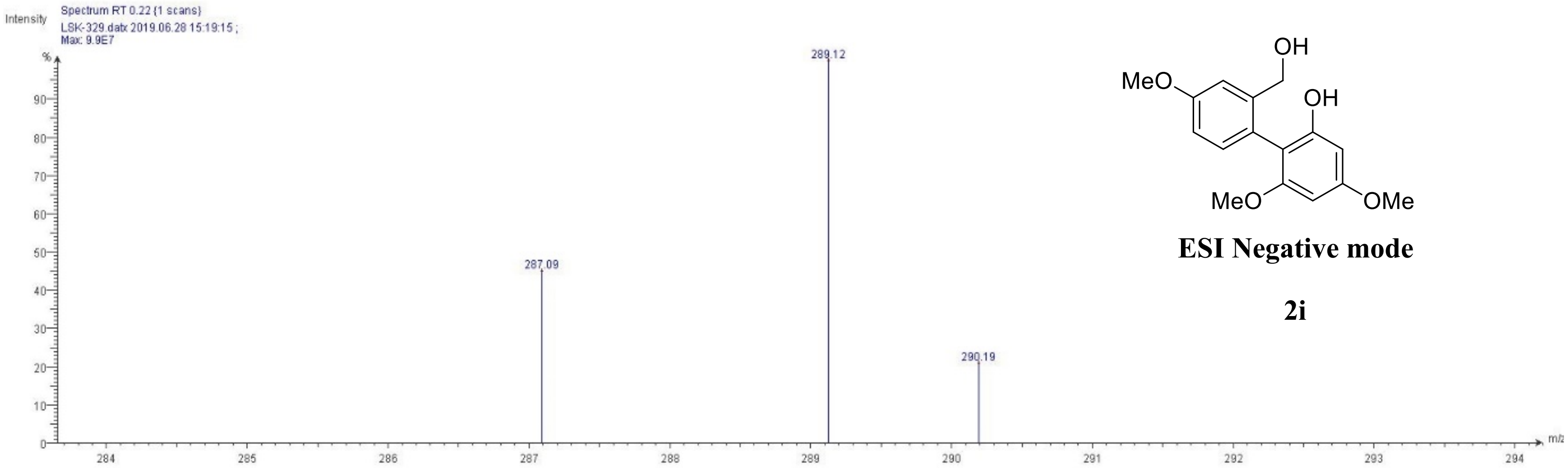

ESI Negative mode

2i

	A	B	C	D	E	F	G	H	I	J	K	L	M	N	O	P
1	f.c (μM)		n1	n2	n3	Blank sample	②S-(SB F4)			③control average	④ activity(%) (②/③F4 *100)			activity aver.	stdev.	stdev/sqrt(count())
2	control		0.33	0.33	0.33	0.04	0.29	0.29	0.29	0.29	100.38	99.52	100.10	100.00	0.44	0.25
3	K.a	50	0.21	0.20	0.18	0.04	0.17	0.15	0.14		57.63	52.49	48.13	52.75	4.76	2.75
4		25	0.23	0.21	0.21	0.04	0.19	0.17	0.17		64.15	59.21	59.04	60.80	2.90	1.68
5		12.5	0.30	0.28	0.27	0.04	0.26	0.24	0.23		87.51	81.54	78.56	82.54	4.56	2.63
6	1c	25	0.15	0.15	0.15	0.04	0.11	0.11	0.11		39.11	38.15	39.11	38.79	0.55	0.32
7		12.5	0.21	0.20	0.19	0.04	0.16	0.16	0.15		56.26	54.82	51.63	54.24	2.37	1.37
8		6.25	0.25	0.26	0.24	0.04	0.21	0.22	0.20		72.69	74.92	66.90	71.50	4.14	2.39
9		3.125	0.30	0.30	0.30	0.04	0.26	0.26	0.26		90.63	90.39	89.16	90.06	0.79	0.46
10		1.5625	0.33	0.32	0.33	0.04	0.28	0.28	0.28		97.39	95.99	97.43	96.94	0.82	0.47
11	1h	6.25	0.15	0.15	0.14	0.04	0.11	0.11	0.10		36.02	36.05	33.21	35.09	1.63	0.94
12		3.125	0.19	0.19	0.19	0.04	0.15	0.15	0.15		51.29	51.29	51.08	51.22	0.12	0.07
13		1.5625	0.25	0.26	0.24	0.04	0.21	0.22	0.20		71.73	75.37	68.89	72.00	3.25	1.88
14		0.78125	0.29	0.29	0.28	0.04	0.25	0.25	0.24		84.32	84.39	81.06	83.26	1.90	1.10
15	2a	25	0.13	0.13	0.13	0.04	0.09	0.09	0.09		30.19	31.29	30.63	30.70	0.55	0.32
16		12.5	0.17	0.18	0.17	0.04	0.13	0.14	0.13		44.19	49.61	45.69	46.50	2.80	1.62
17		6.25	0.24	0.24	0.24	0.04	0.20	0.20	0.20		67.75	68.34	68.78	68.29	0.52	0.30
18		3.125	0.25	0.30	0.31	0.04	0.21	0.26	0.27		71.94	89.71	92.97	84.87	11.32	6.53
19		1.5625	0.33	0.33	0.33	0.04	0.29	0.29	0.29		99.73	99.28	98.94	99.31	0.40	0.23

	B	C	D	E	F	G	H	I	J	K	L	M
21		f.c (μM)	Inhibition (%)			inhibition (aver.)	IC50			IC50(aver)	stdev.	ev/sqrt(count())
22	K.a	50	43.81	48.83	53.08	48.57	54.82	47.86	43.19	48.62	5.85	3.38
23		25	37.46	42.27	42.44	40.72						
24		12.5	14.68	20.50	23.41	19.53						
25	1c	25	60.89	61.85	60.89	61.21	18.46	18.18	17.62	18.09	0.42	0.25
26		12.5	43.74	45.18	48.37	45.76						
27		6.25	27.31	25.08	33.10	28.50						
28		3.125	9.37	9.61	10.84	9.94						
29		1.5625	2.61	4.01	2.57	3.06						
30	1h	6.25	63.98	63.95	66.79	64.91	4.20	4.27	3.94	4.14	0.17	0.10
31		3.125	48.71	48.71	48.92	48.78						
32		1.5625	28.27	24.63	31.11							
33		0.78125	15.68	15.61	18.94	16.74						
34	2a	25	69.81	68.71	69.37	69.30	14.68	16.02	15.69	15.46	0.70	0.40
35		12.5	55.81	50.39	54.31	53.50						
36		6.25	32.25	31.66	31.22	31.71						
37		3.125	28.06	10.29	7.03	15.13						
38		1.5625	0.27	0.72	1.06	0.69						

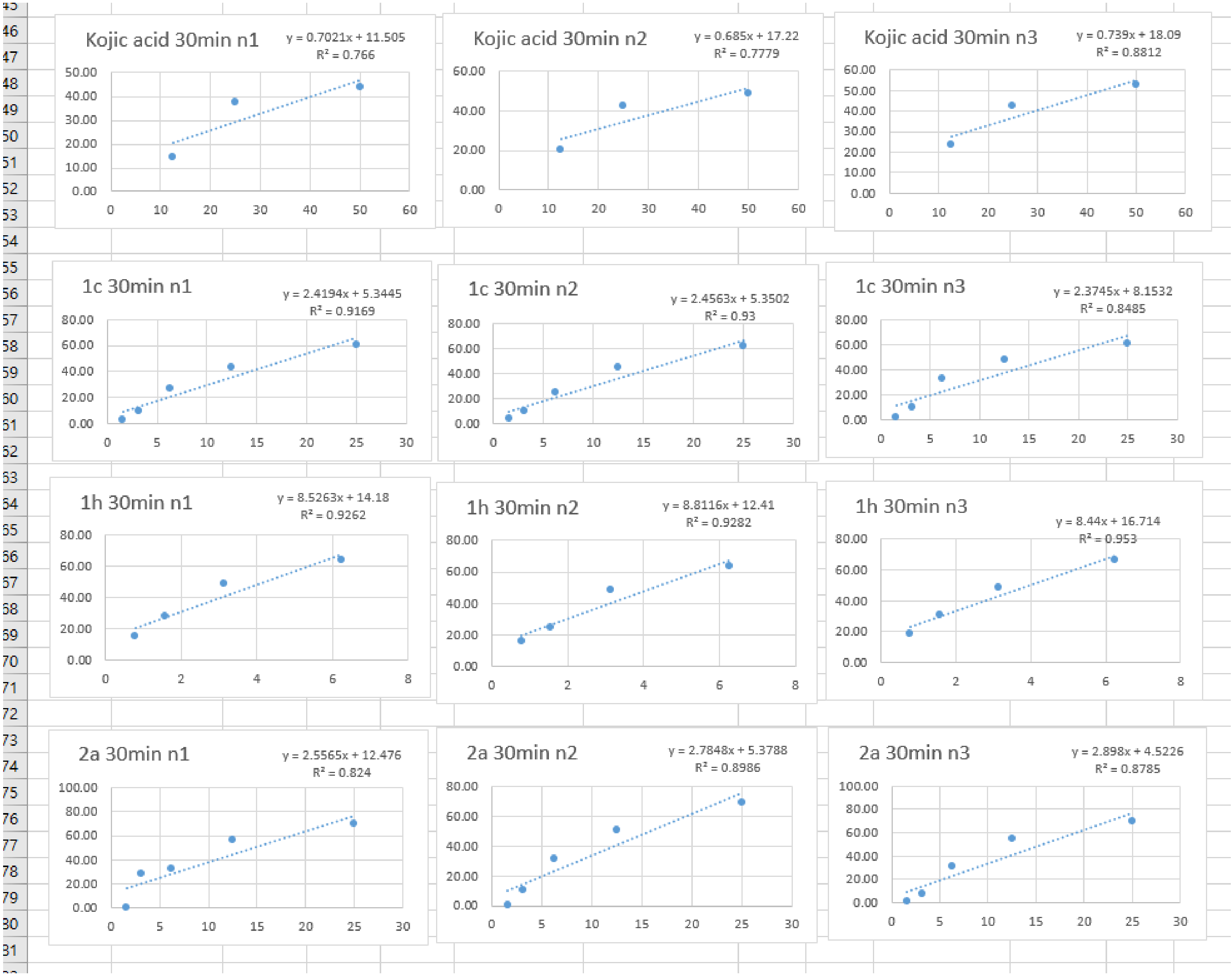
Kojic acid 30min n1
y = 0.7021x + 11.505
R² = 0.766
Kojic acid 30min n2
y = 0.685x + 17.22
R² = 0.7779
Kojic acid 30min n3
y = 0.739x + 18.09
R² = 0.8812
1c 30min n1
y = 2.4194x + 5.3445
R² = 0.9169
1c 30min n2
y = 2.4563x + 5.3502
R² = 0.93
1c 30min n3
y = 2.3745x + 8.1532
R² = 0.8485
1h 30min n1
y = 8.5263x + 14.18
R² = 0.9262
1h 30min n2
y = 8.8116x + 12.41
R² = 0.9282
1h 30min n3
y = 8.44x + 16.714
R² = 0.953
2a 30min n1
y = 2.5565x + 12.476
R² = 0.824
2a 30min n2
y = 2.7848x + 5.3788
R² = 0.8986
2a 30min n3
y = 2.898x + 4.5226
R² = 0.8785
